# Correction: The sockeye salmon genome, transcriptome, and analyses identifying population defining regions of the genome

**DOI:** 10.1371/journal.pone.0262189

**Published:** 2021-12-30

**Authors:** Kris A. Christensen, Eric B. Rondeau, David R. Minkley, Dionne Sakhrani, Carlo A. Biagi, Anne-Marie Flores, Ruth E. Withler, Scott A. Pavey, Terry D. Beacham, Theresa Godin, Eric B. Taylor, Michael A. Russello, Robert H. Devlin, Ben F. Koop

This article [1] cites a *PLOS ONE* article that was retracted on February 9, 2021 [2, 3] because the sample used for genome analysis was found to have been misidentified. The genomic assembly reported in [2] is still available at GenBank, but it has been relabelled as an unclassified species in the *Salvelinus* genus, or *Salvelinus sp*. (GenBank accession: GCF_002910315.2).

The draft genome reported in [2] was one of six reference species used to confirm synteny placement of scaffolds during assembly of the sockeye salmon genome in this 2020 article [1]. The authors and an Academic Editor concluded that the retraction [3] and the species identity of the genome reported in [2] do not impact the validity or reliability of this article’s [1] results and conclusions as multiple species and multiple lines of evidence were used to confirm synteny assessments.

The retracted article [2] was also cited as a source for methods used in the 2020 article [1]. As a result, the authors have provided the relevant Python scripts via GitHub and the Data Availability statement for this article [1] is as follows: Raw data has been deposited to the National Center for Biotechnology Information (NCBI) under BioProject PRJNA530256 (https://www.ncbi.nlm.nih.gov/bioproject/PRJNA530256/). Custom scripts and sample information can be found in supplemental files and via GitHub: https://github.com/KrisChristensen/OrthologyBetweenSpecies.
