# Supplementary figures and images for: The sockeye salmon genome, transcriptome, and analyses identifying population defining regions of the genome
Source: PLoS One. 2020 Oct 29;15(10):e0240935. doi: 10.1371/journal.pone.0240935 (PMC7595290; doi:10.1371/journal.pone.0240935)

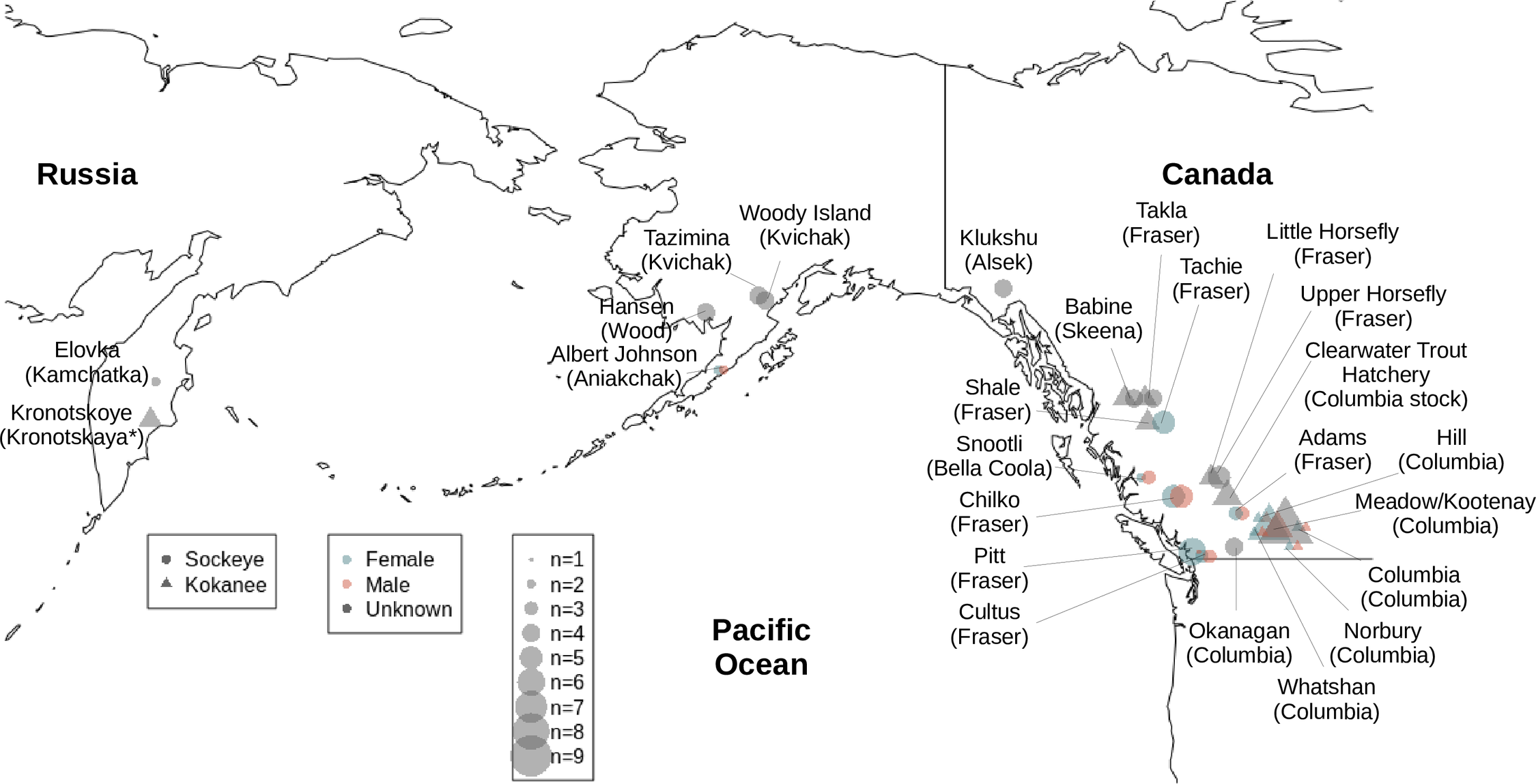

Supplement: S1 Fig — Map generated with the maps library in R [154]. (TIF) [file pone.0240935.s001.tif]

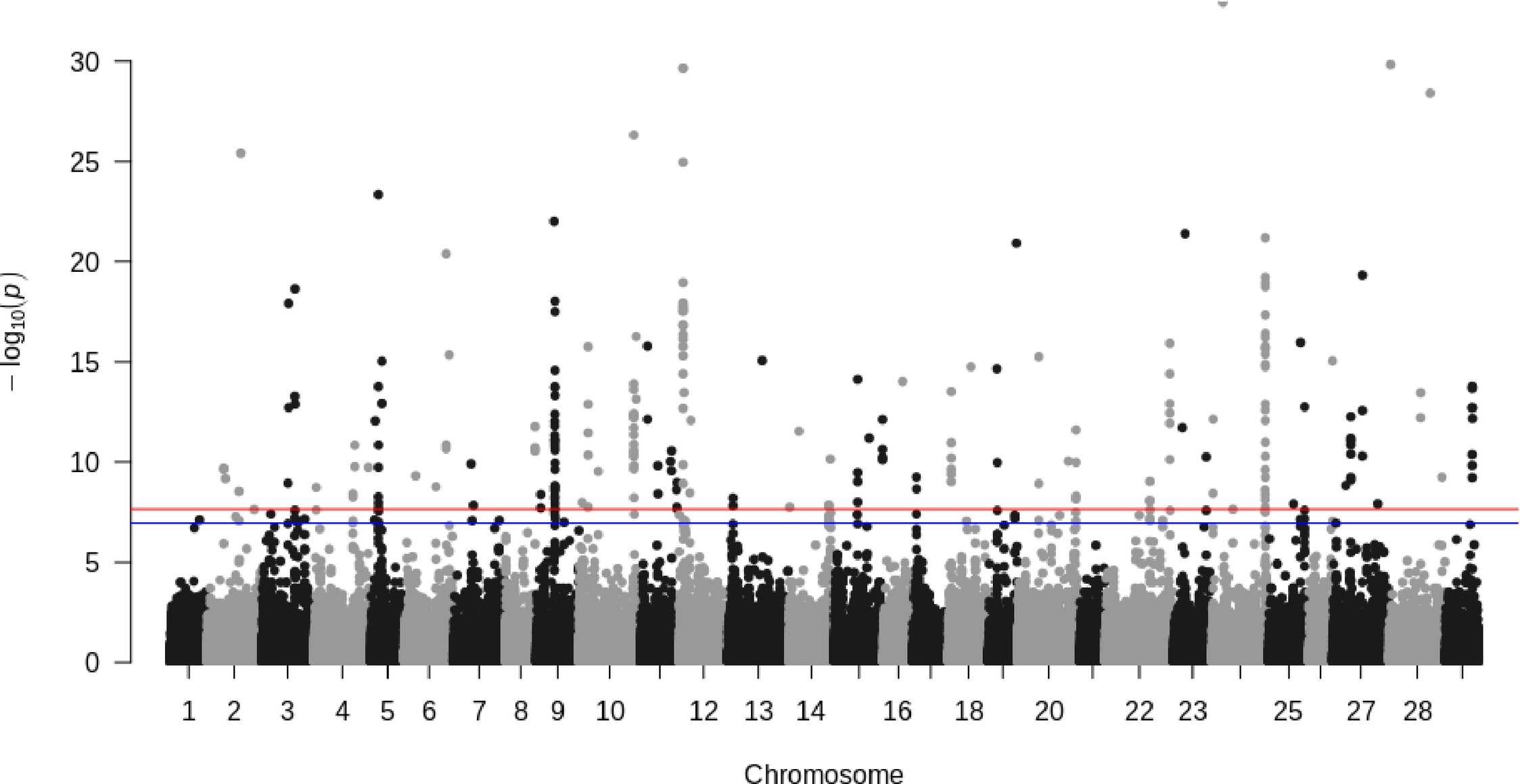

Supplement: S2 Fig — A Manhattan plot where eigenvalues from the DAPC analysis were used to identify regions of the genome with ancestry informative genes (e.g. under selection) between groups 1 and 2. The red horizontal line is the threshold of significance for ɑ = 0.01 after Bonferroni correction.The blue line is for ɑ = 0.05. (TIF) [file pone.0240935.s002.tif]

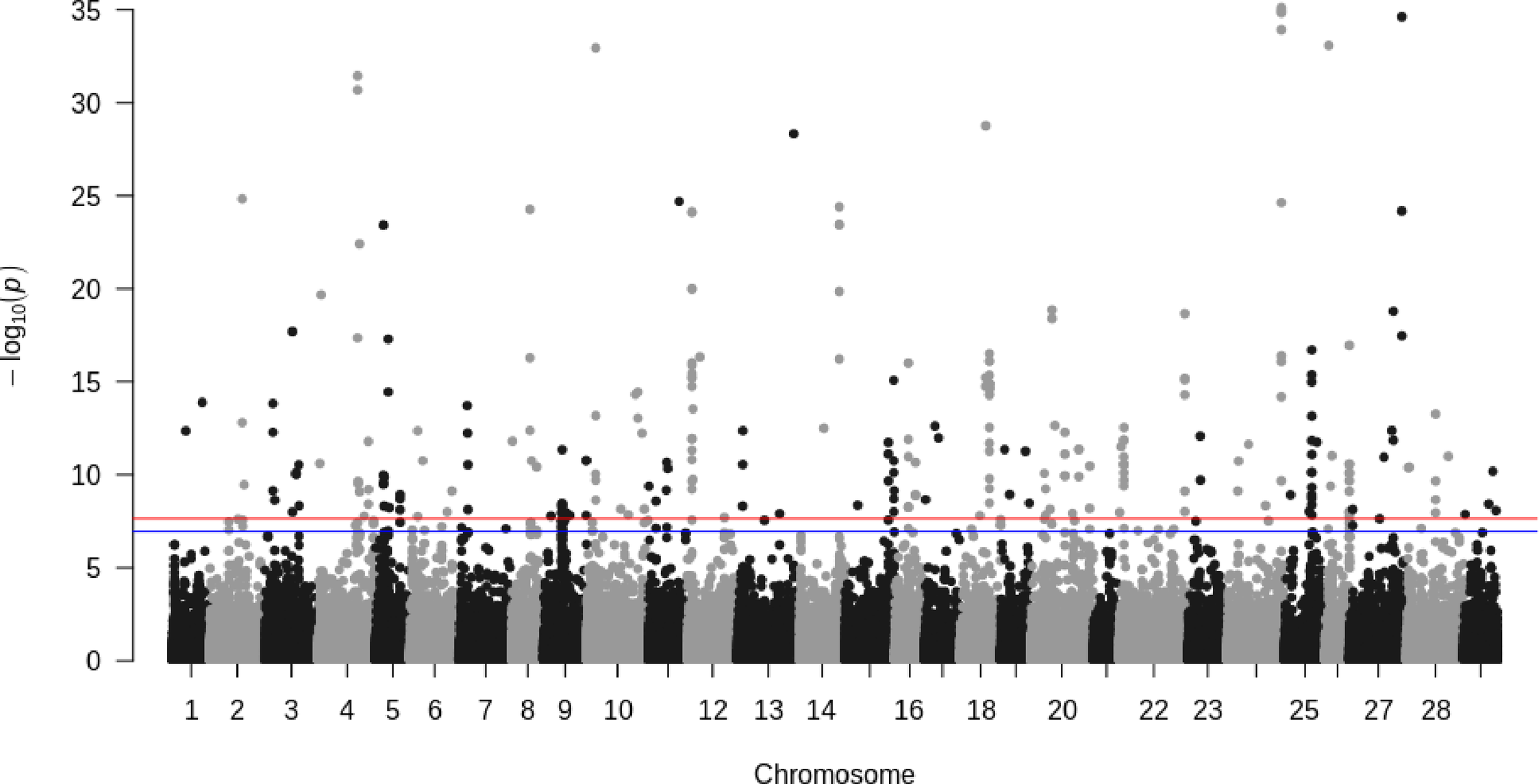

Supplement: S3 Fig — A Manhattan plot where eigenvalues from the DAPC analysis were used to identify regions of the genome with ancestry informative genes (e.g. under selection) between groups 1 and 3. The red horizontal line is the threshold of significance for ɑ = 0.01 after Bonferroni correction. The blue line is for ɑ = 0.05. (TIF) [file pone.0240935.s003.tif]

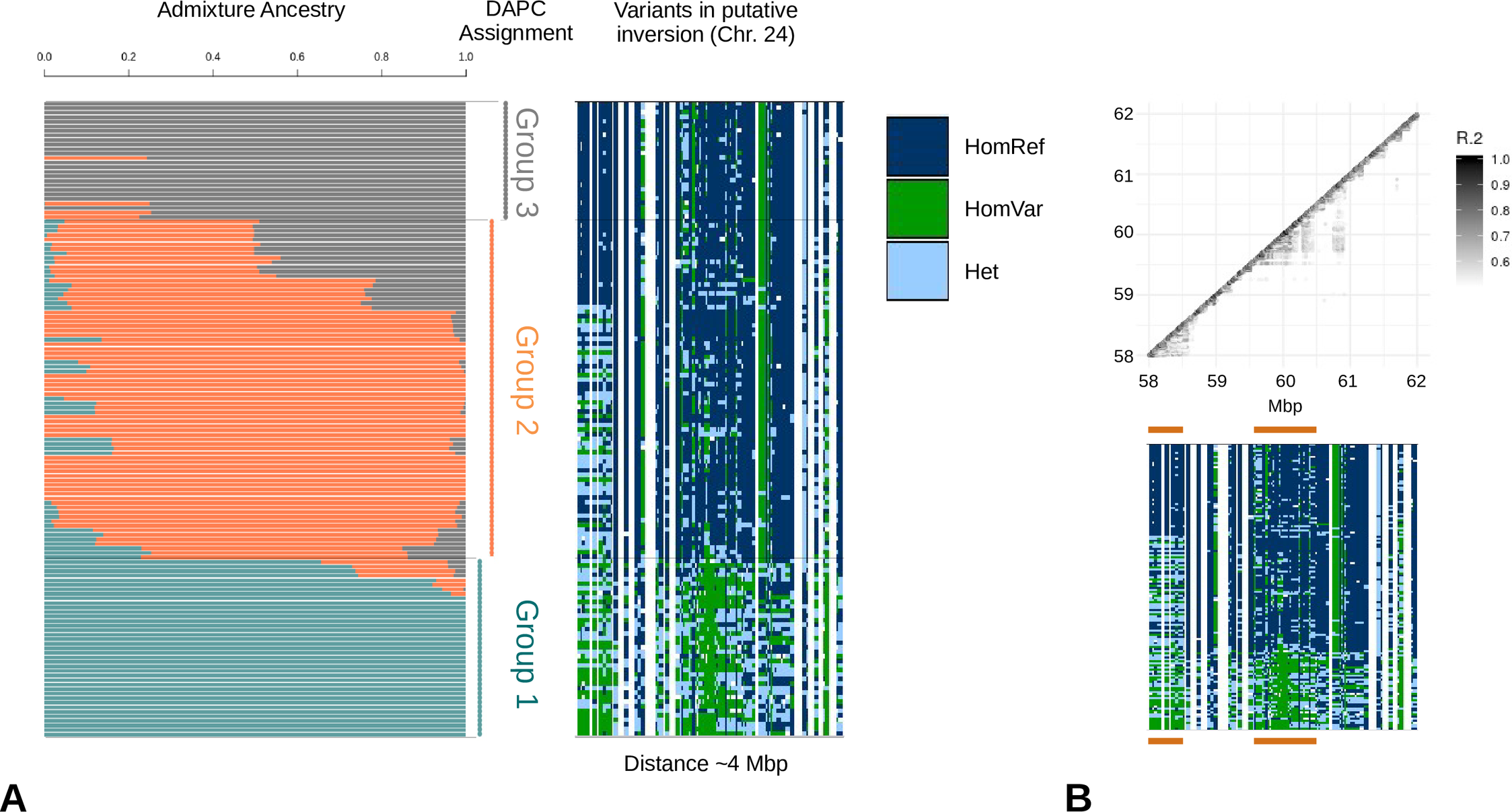

Supplement: S4 Fig — A) On the left of this figure is the admixture ancestry plot with the DAPC group assignments. On the right, is a screenshot of chromosome 24 from IGV from 58 Mbp—62 Mbp (only variants with r2 values > = 0.3 with the variant with the lowest p-value from the eigenGWA in this peak are shown). This region of the genome was found from an eigenGWA to be associated with inferred population structure between DAPC groups 1 and 2. The dark blue genotypes are homozygous for the reference allele (HomRef), the green genotypes are homozygous for an alternative allele (HomVar), and the light blue are heterozygous (Het). B) A scatterplot of variants with r2 values > = 0.5 on the top shows areas with high LD. Below is a smaller version of the genotypes with the putative inversions highlighted. (TIF) [file pone.0240935.s004.tif]

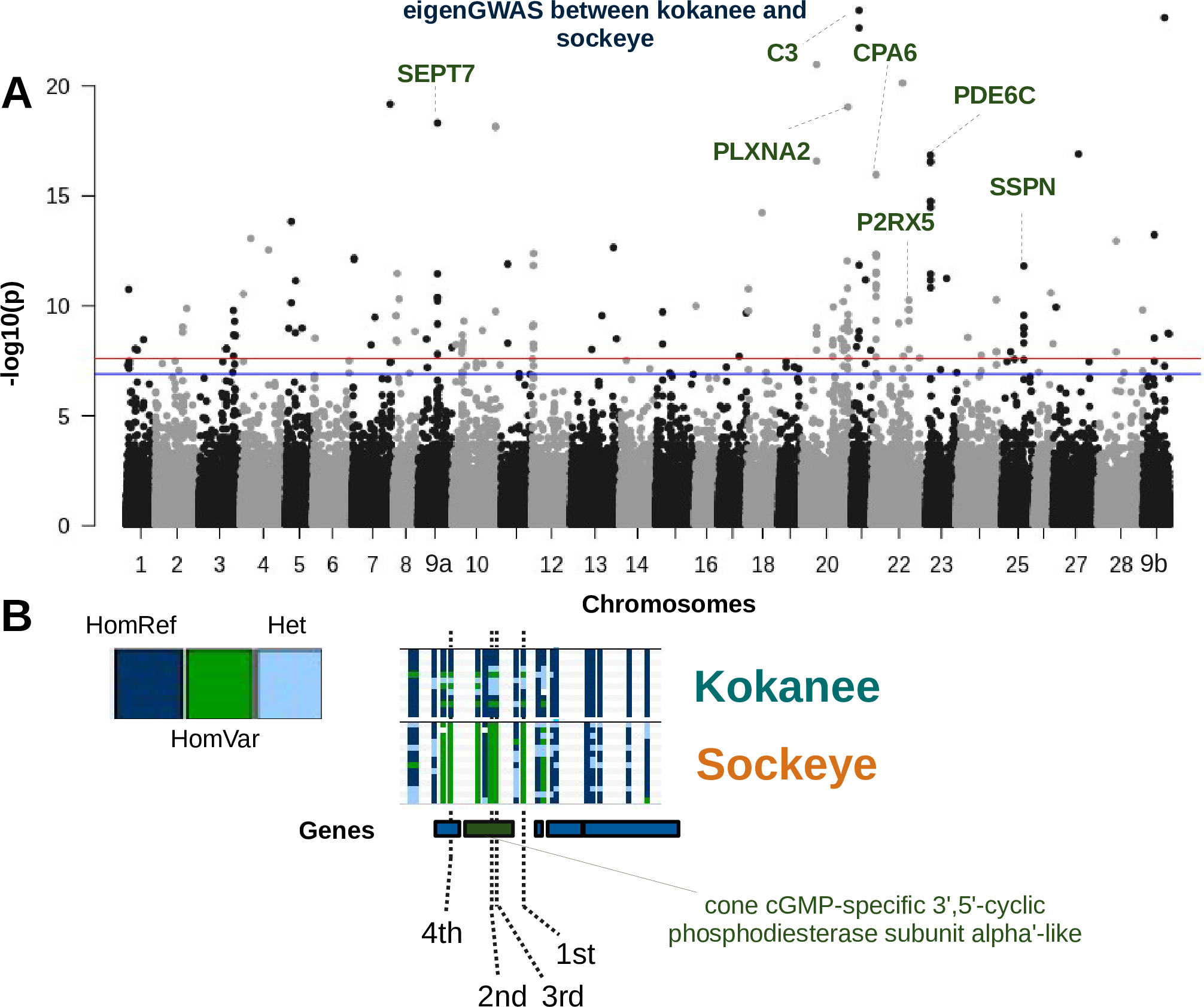

Supplement: S5 Fig — A) The eigenGWA is shown between Fraser River sockeye salmon (n = 14) and kokanee (n = 12) with putative genes highlighted at the peaks (with at least 5 variants with LD). The red line represents a Bonferroni correction at ɑ = 0.01 and after correction for the genomic inflation factor. The blue line represents a Bonferroni correction at ɑ = 0.05 and was chosen as the minimum value of significance. B) An IGV plot of all the variants used in the eigenGWA for the region around the peak on chromosome 23. The genotypes are: dark blue—homozygous reference, green—homozygous alternative, and light blue—heterozygous. The top IGV plot is the kokanee used in this analysis and the sockeye are below. Below the IGV plot, thick lines represent NCBI annotated genes in this region. The putative ancestry informative gene is highlighted in green and named. The variants with the lowest p-values from the eigenGWA are shown as dotted-lines (1st represents the variant with the lowest p-value, 2nd represents the variant with the second lowest p-value, etc.). The p-values, in combination with the genotypes, were used to identify the most likely ancestry informative gene in this region. (TIF) [file pone.0240935.s005.tif]

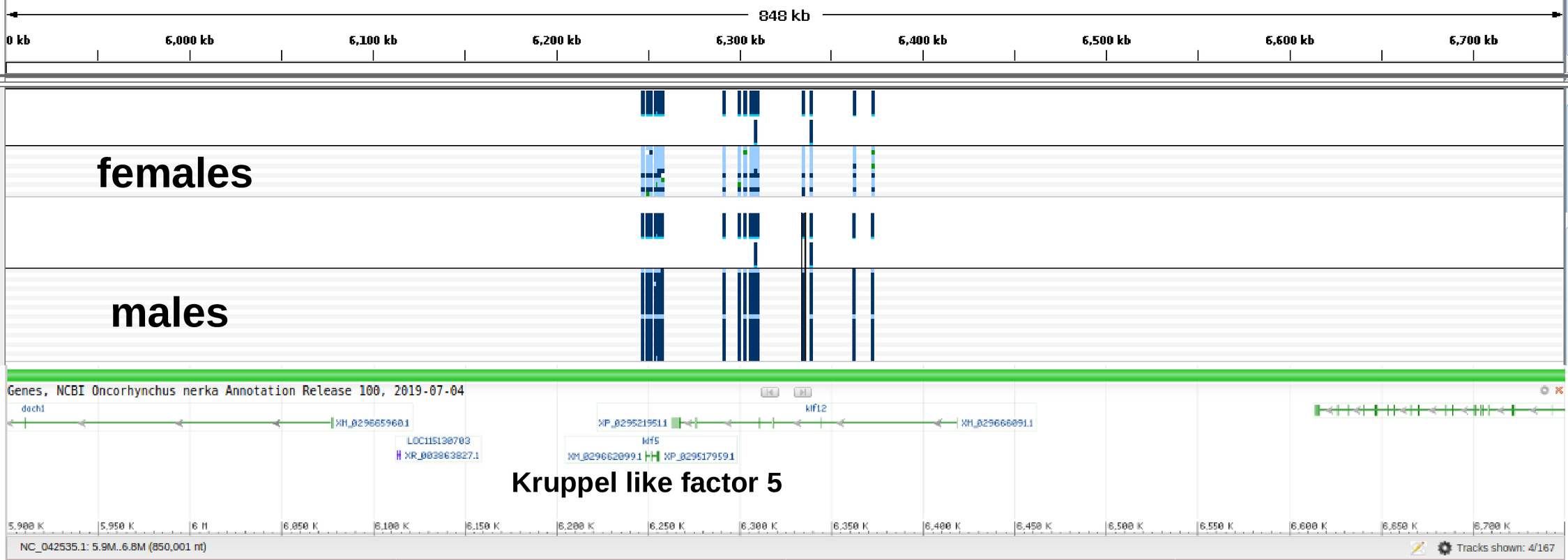

Supplement: S6 Fig — Variants on chromosome 1 (NC_042535.1) shown in IGV with the female variants on top and the male variants on the bottom. The variant with the greatest association was found in the 3’ UTR of the krüppel-like factor 5 gene. (TIF) [file pone.0240935.s006.tif]

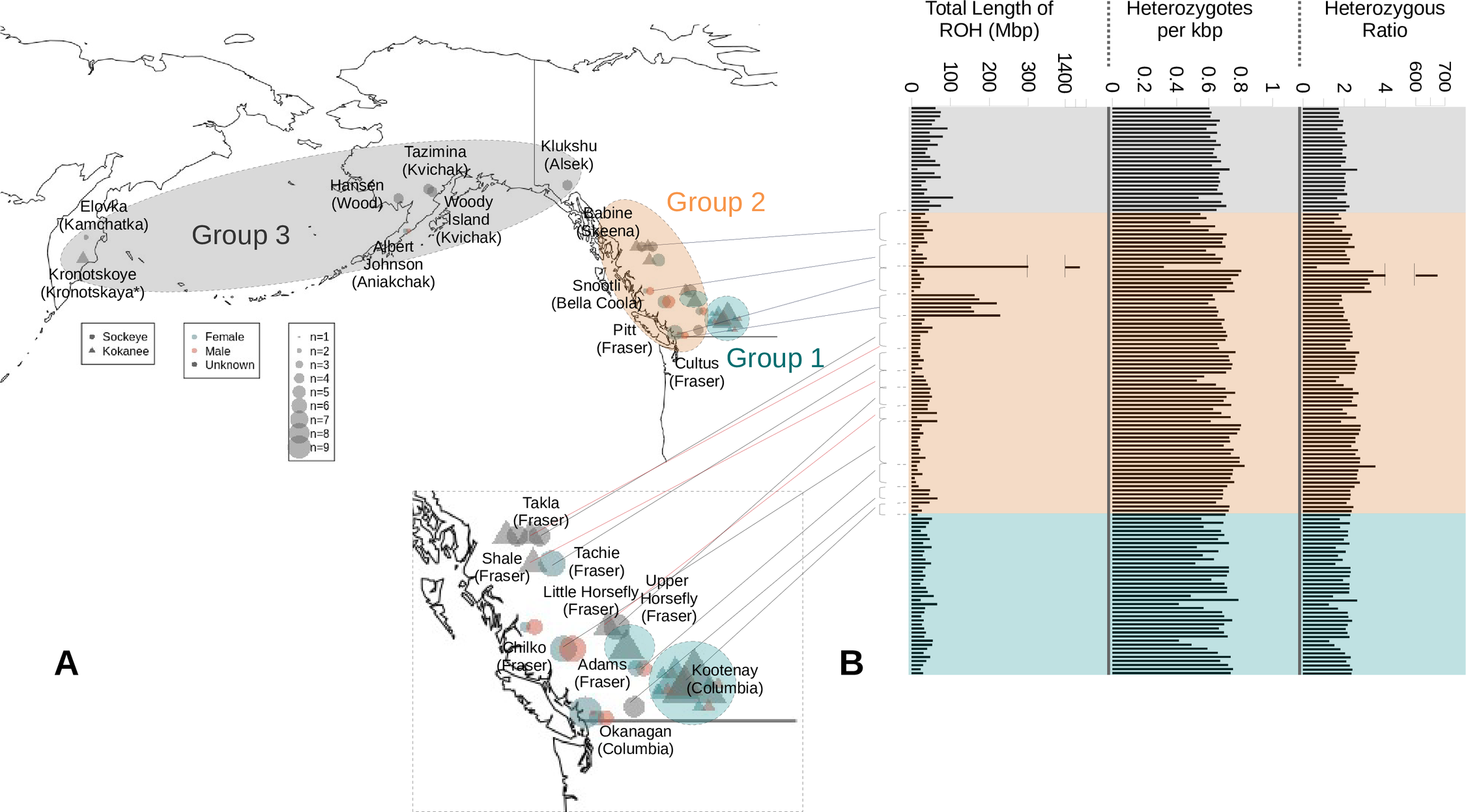

Supplement: S7 Fig — A) A map of the sampling sites. B) Three measures of individual genomic diversity: 1) total length of runs of homozygosity, 2) heterozygous genotypes per kbp, and 3) heterozygous ratio. (TIF) [file pone.0240935.s007.tif]

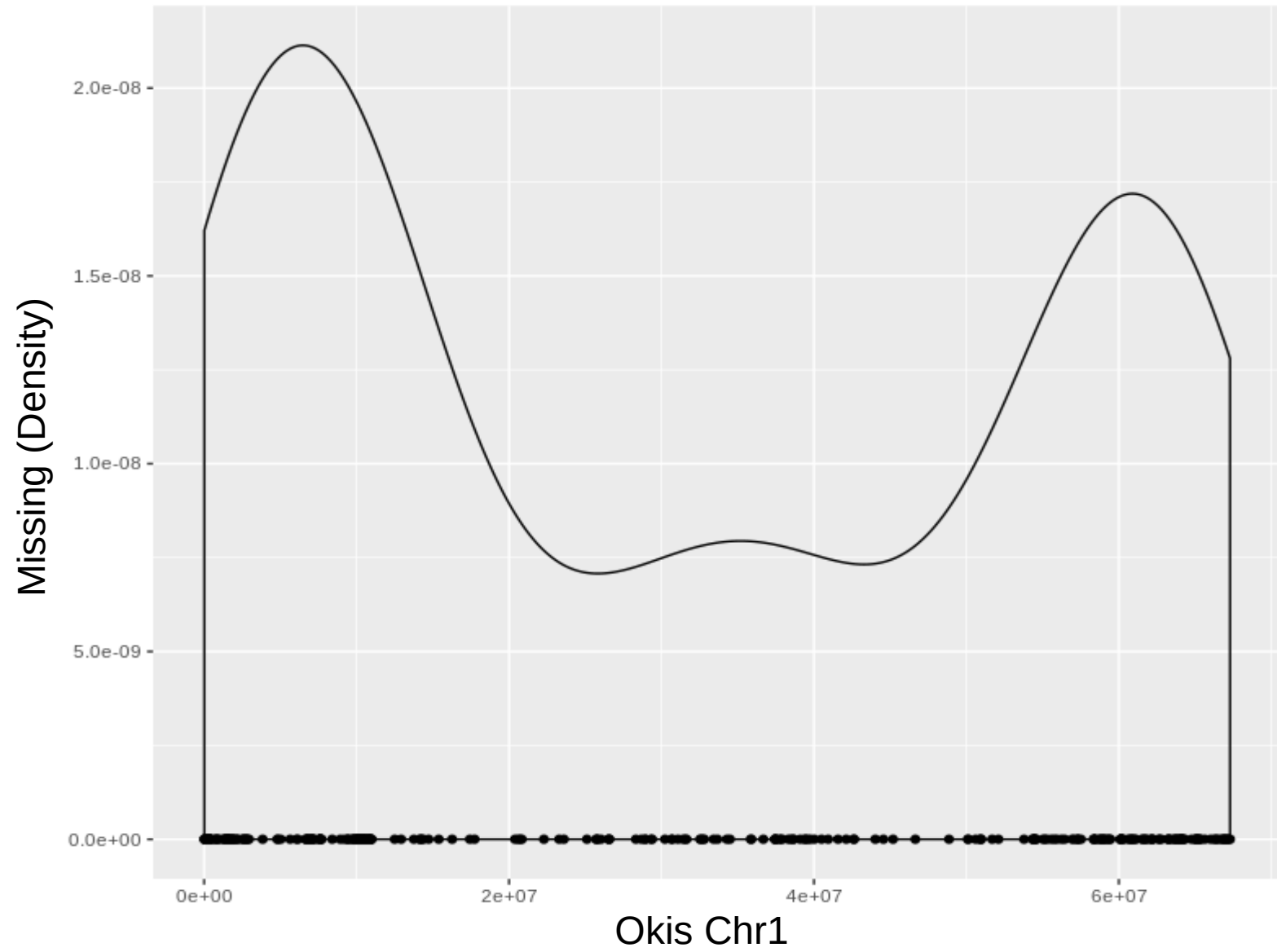

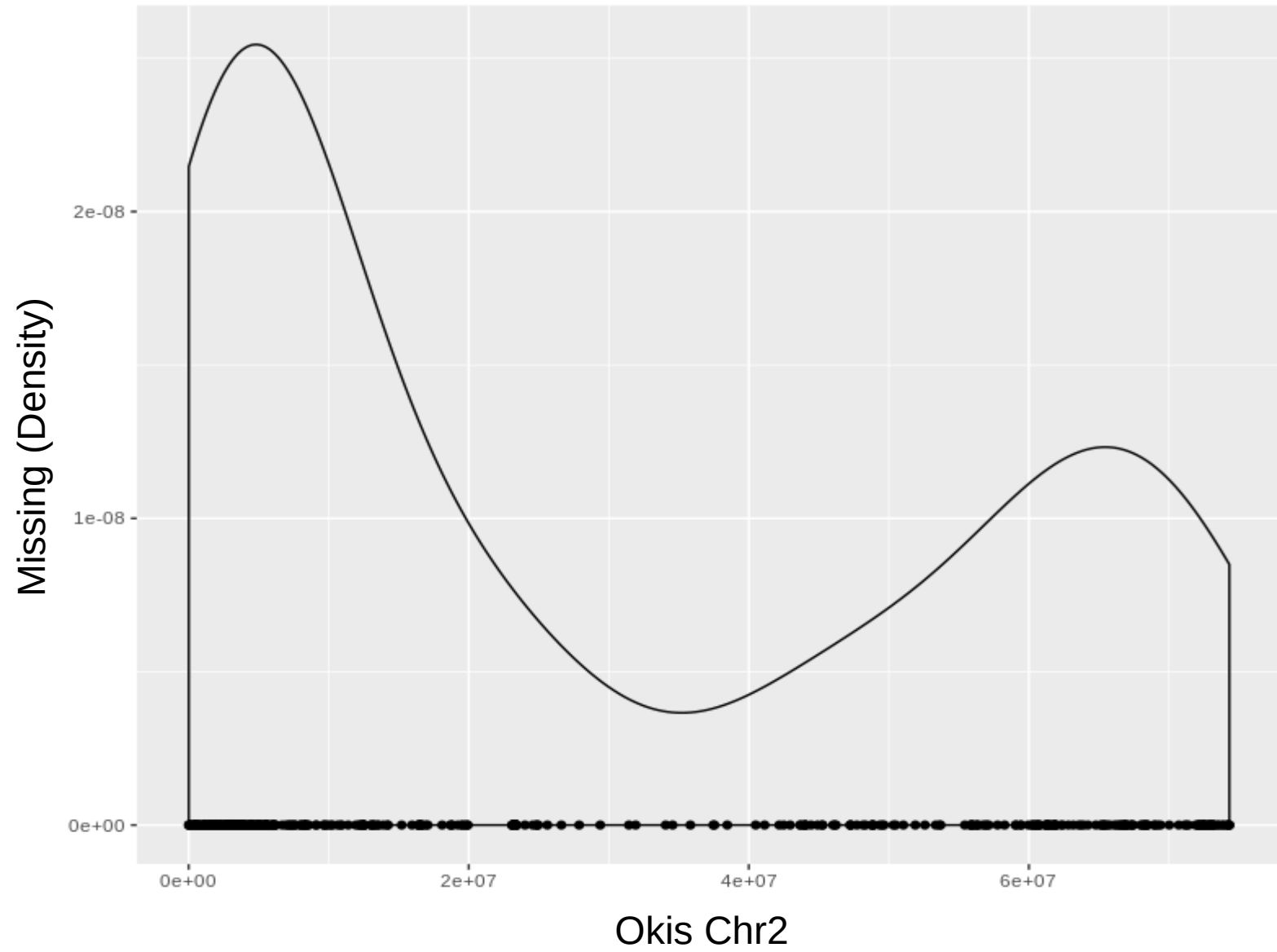

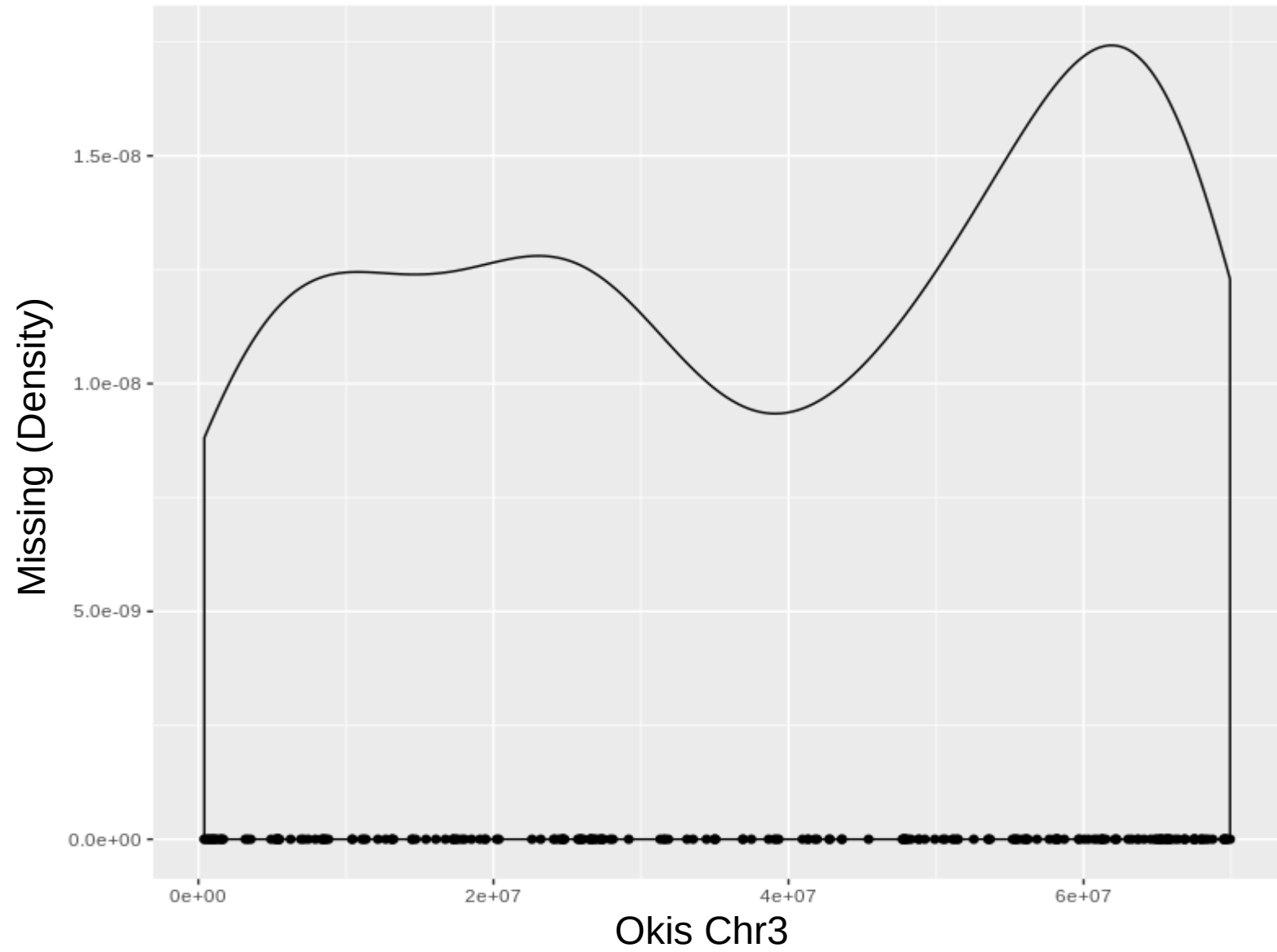

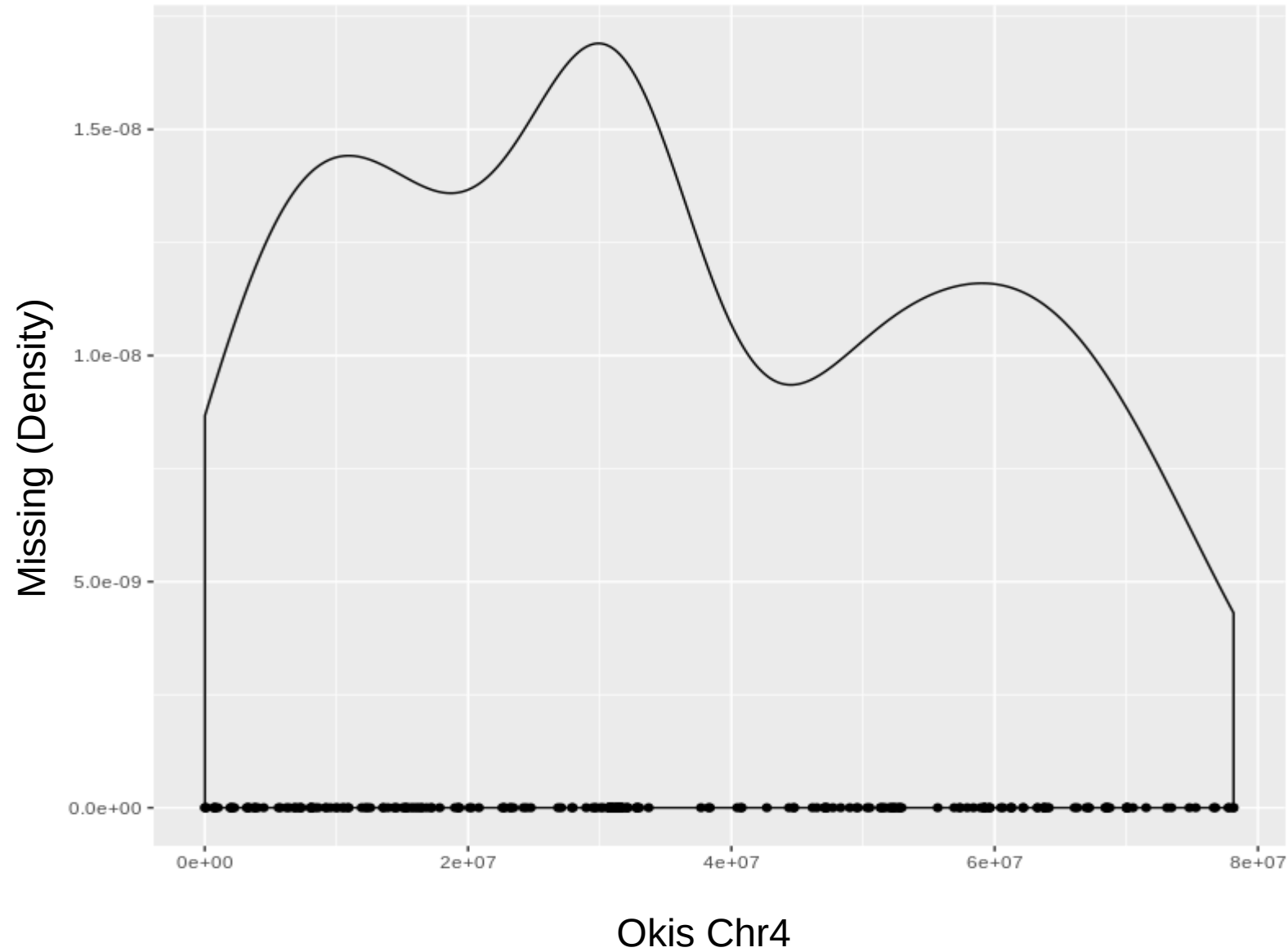

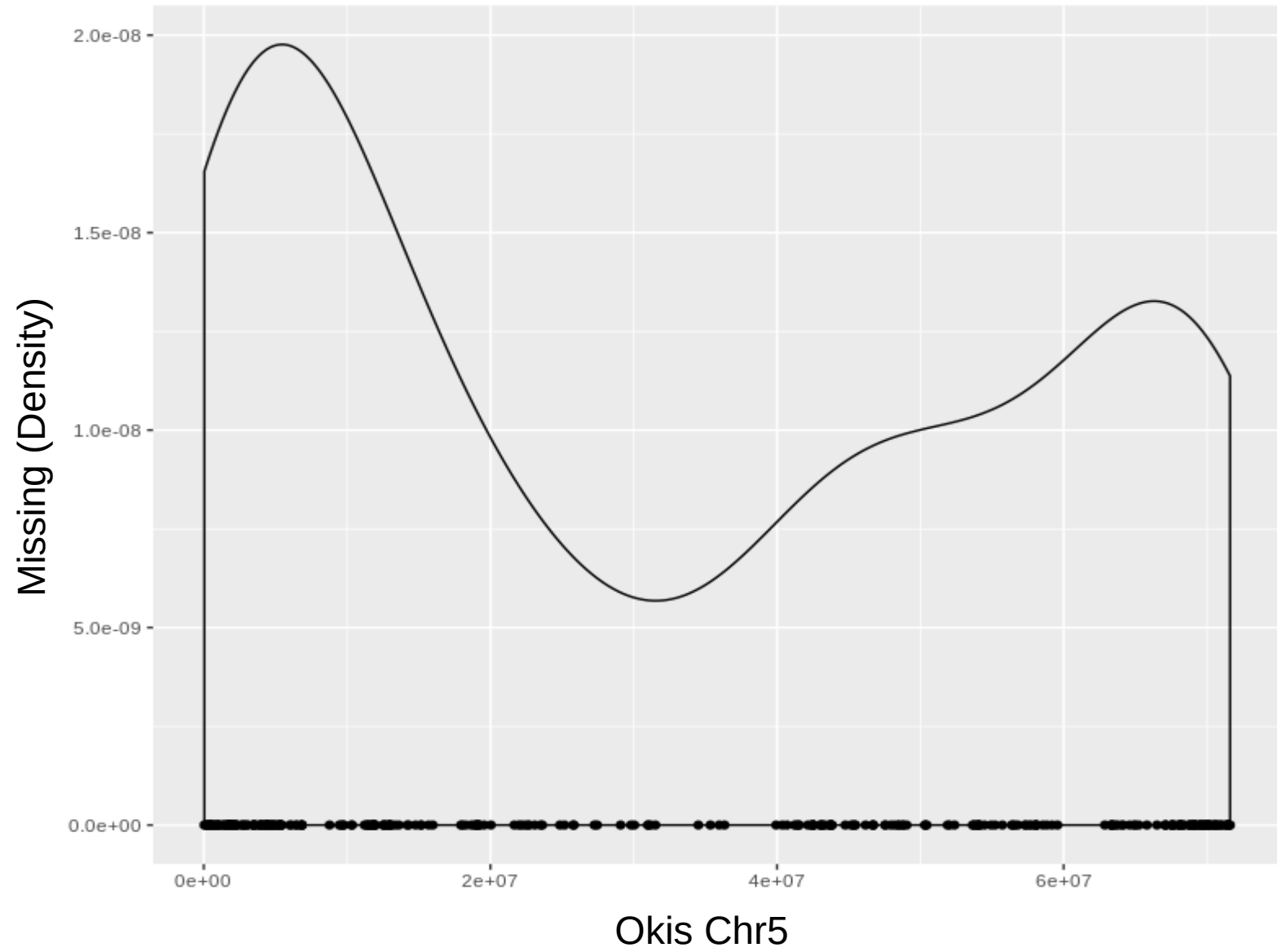

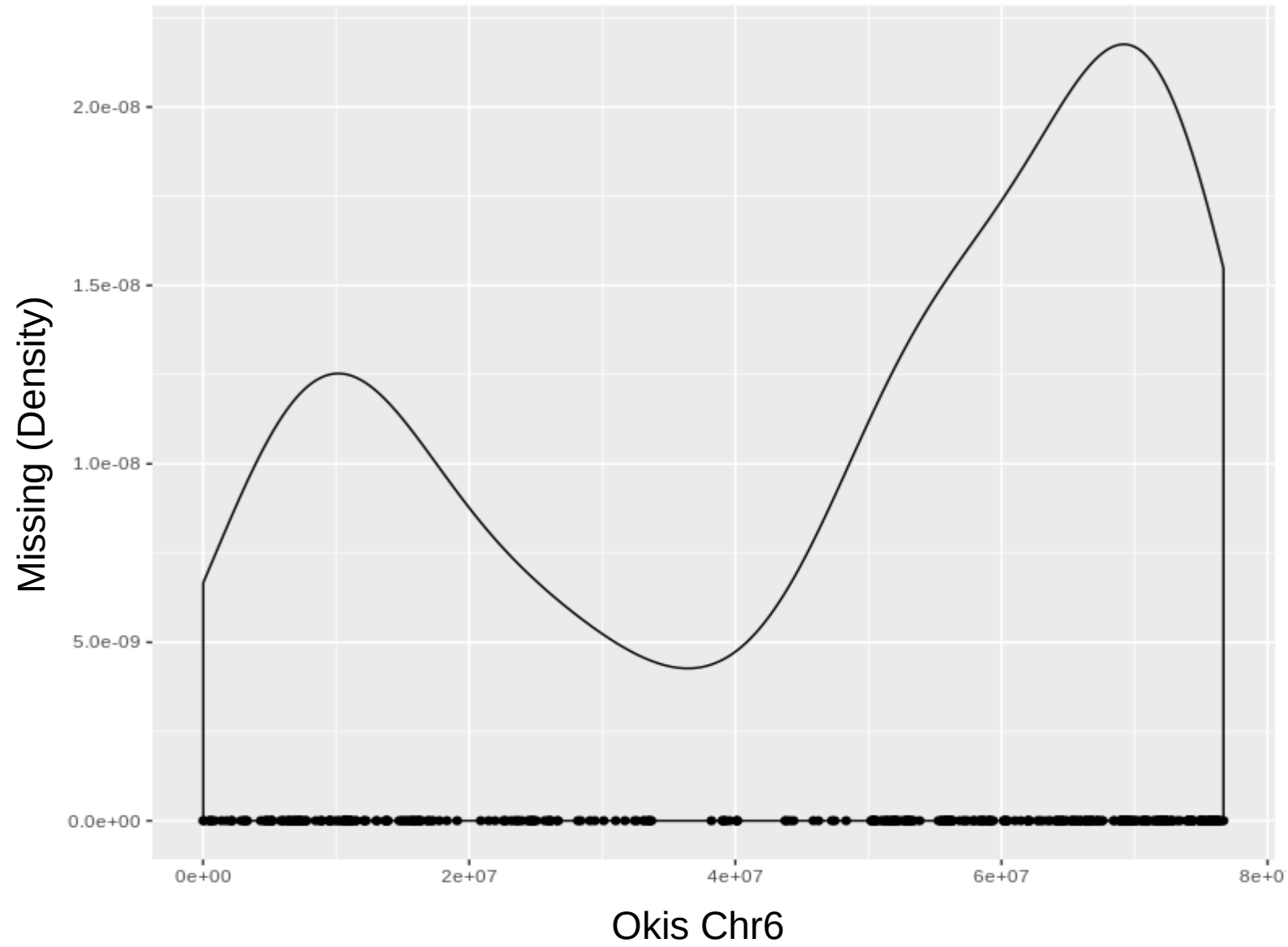

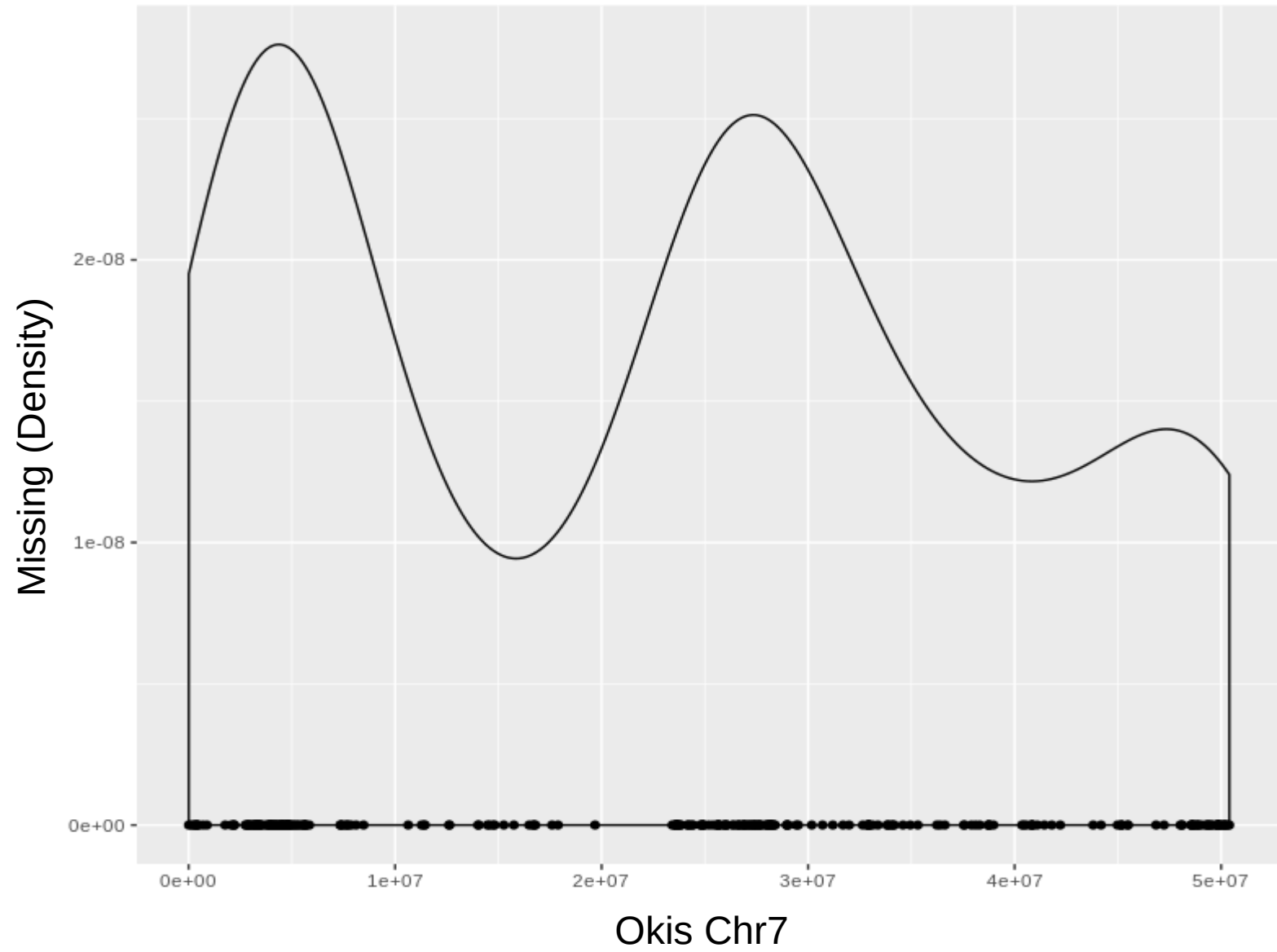

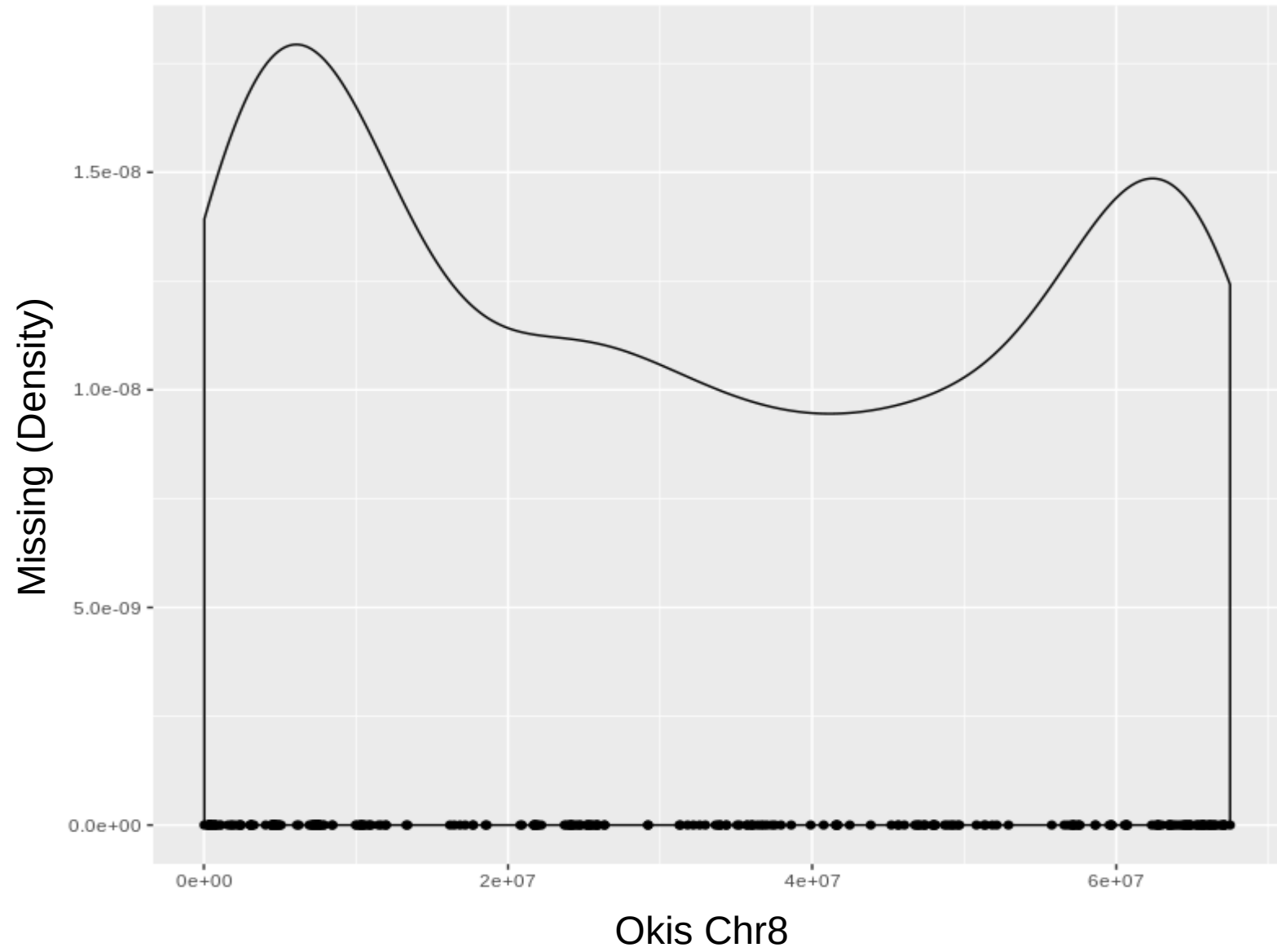

Missing (Density)

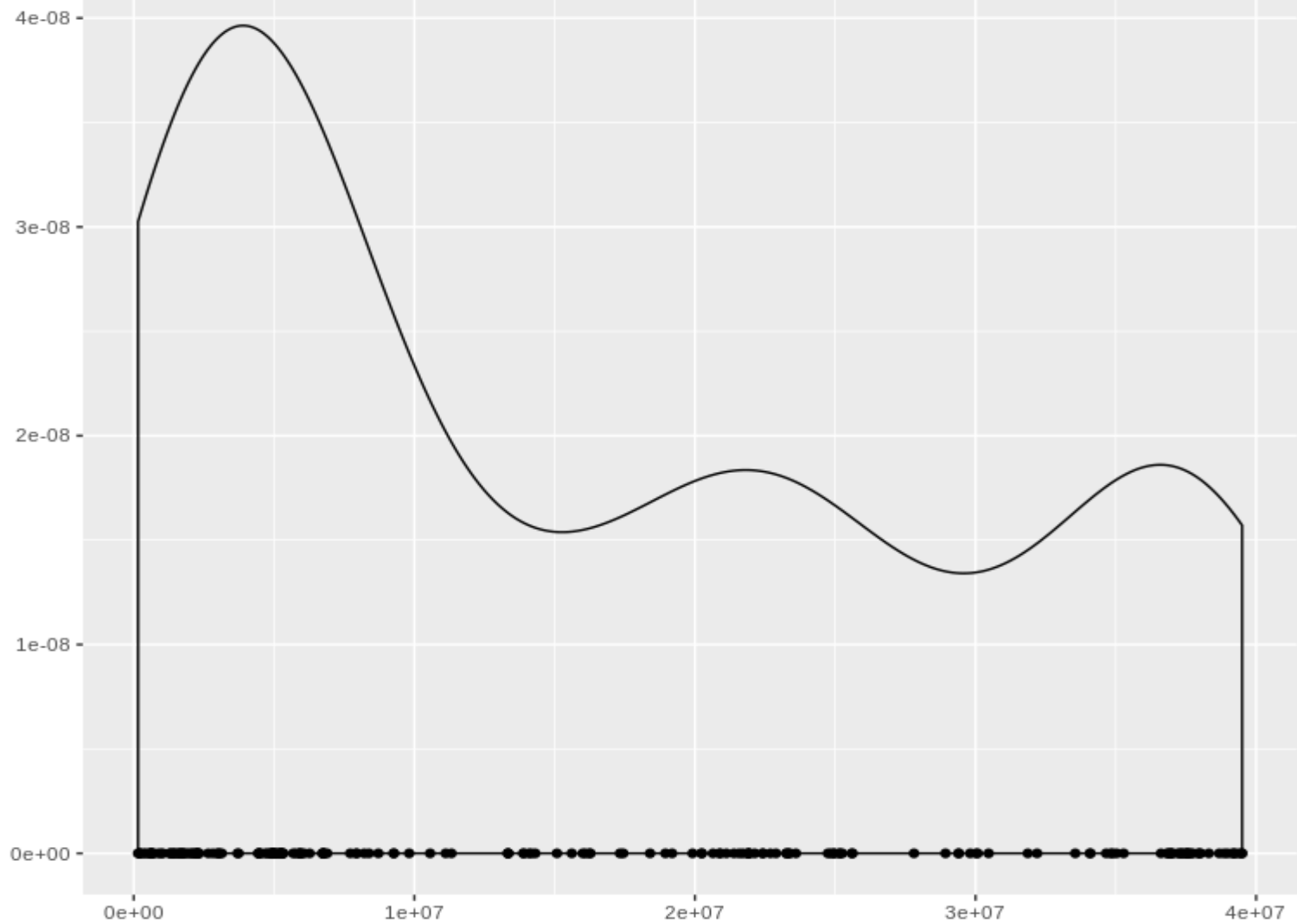

Okis Chr9

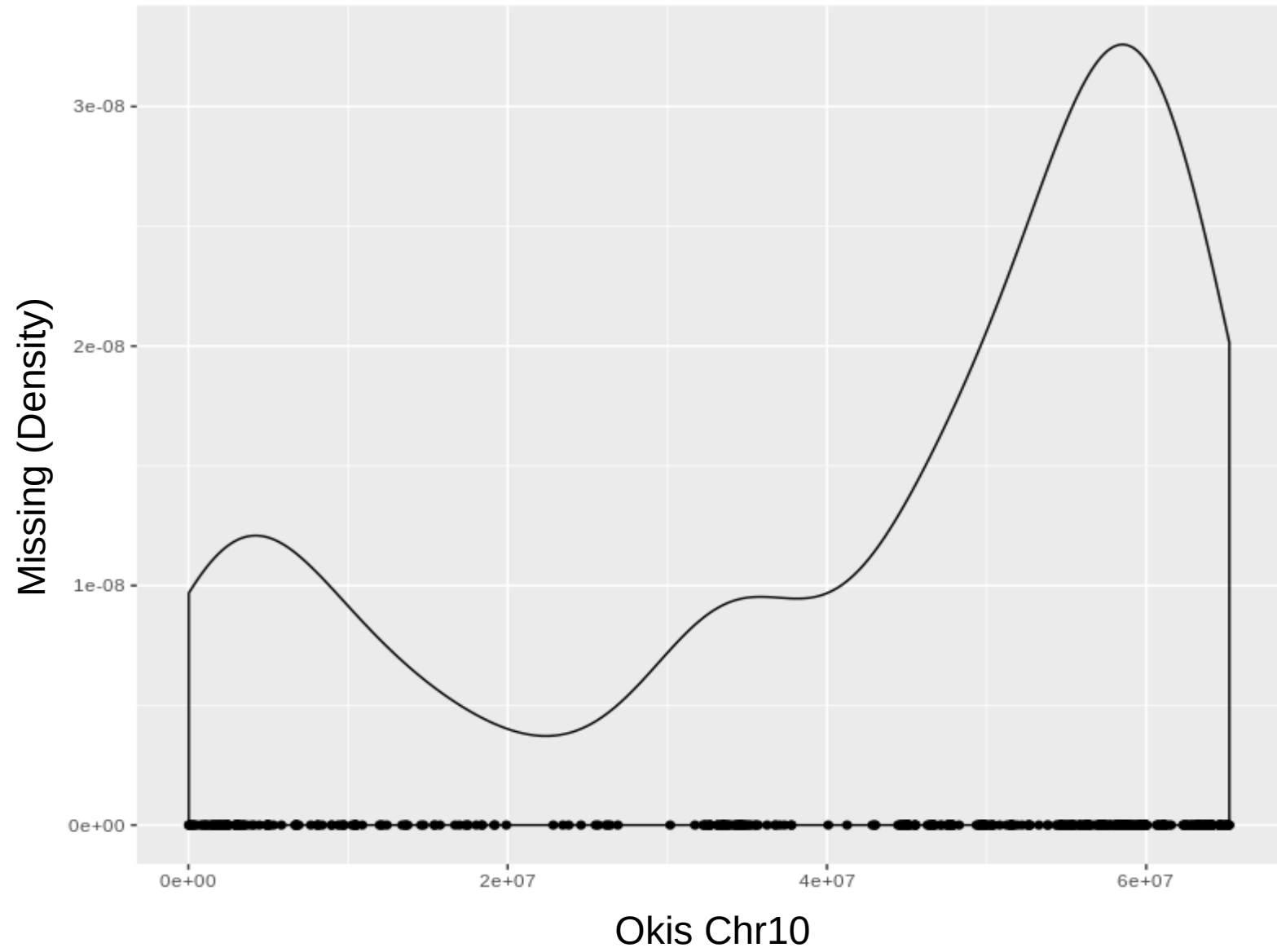

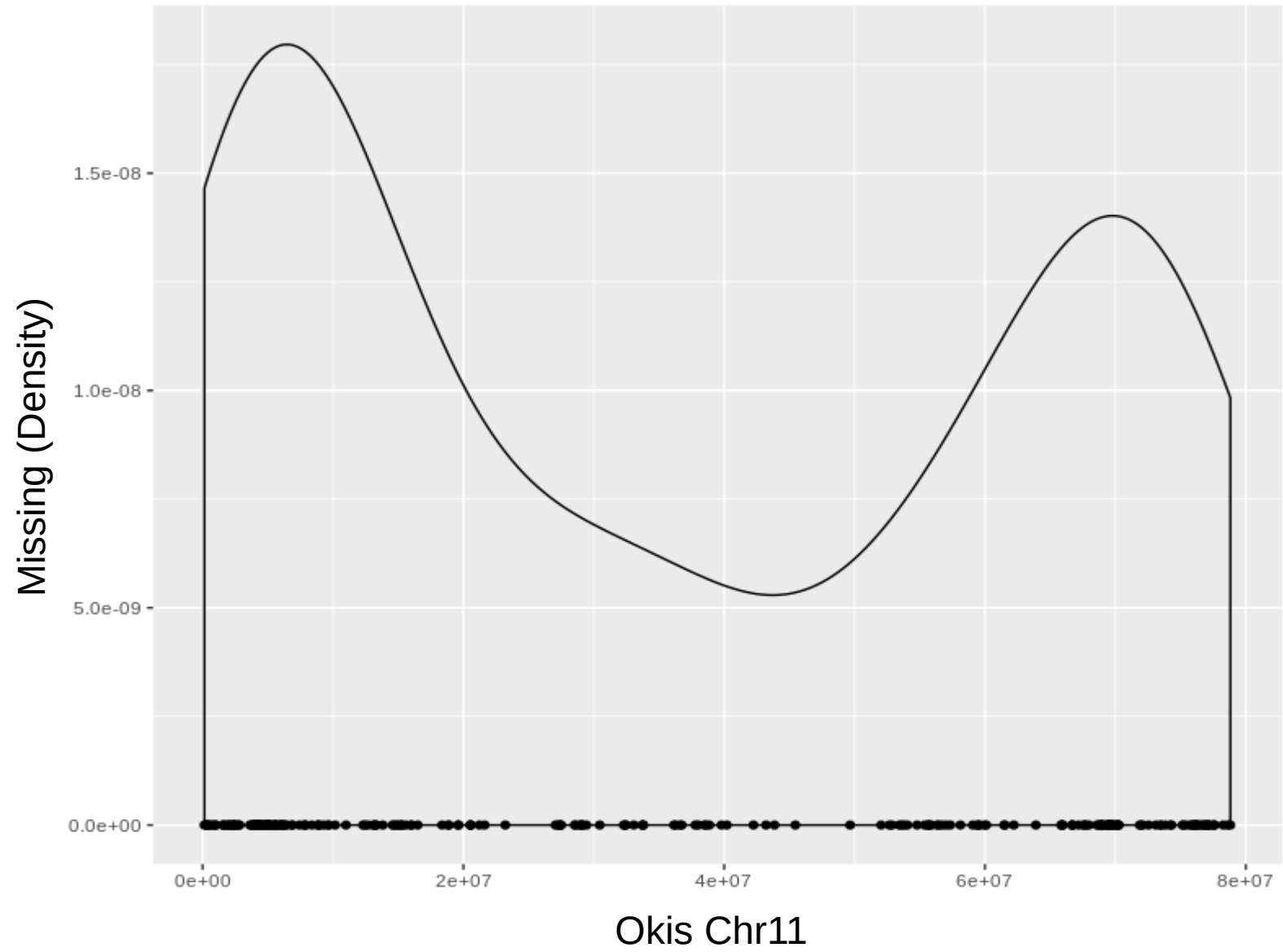

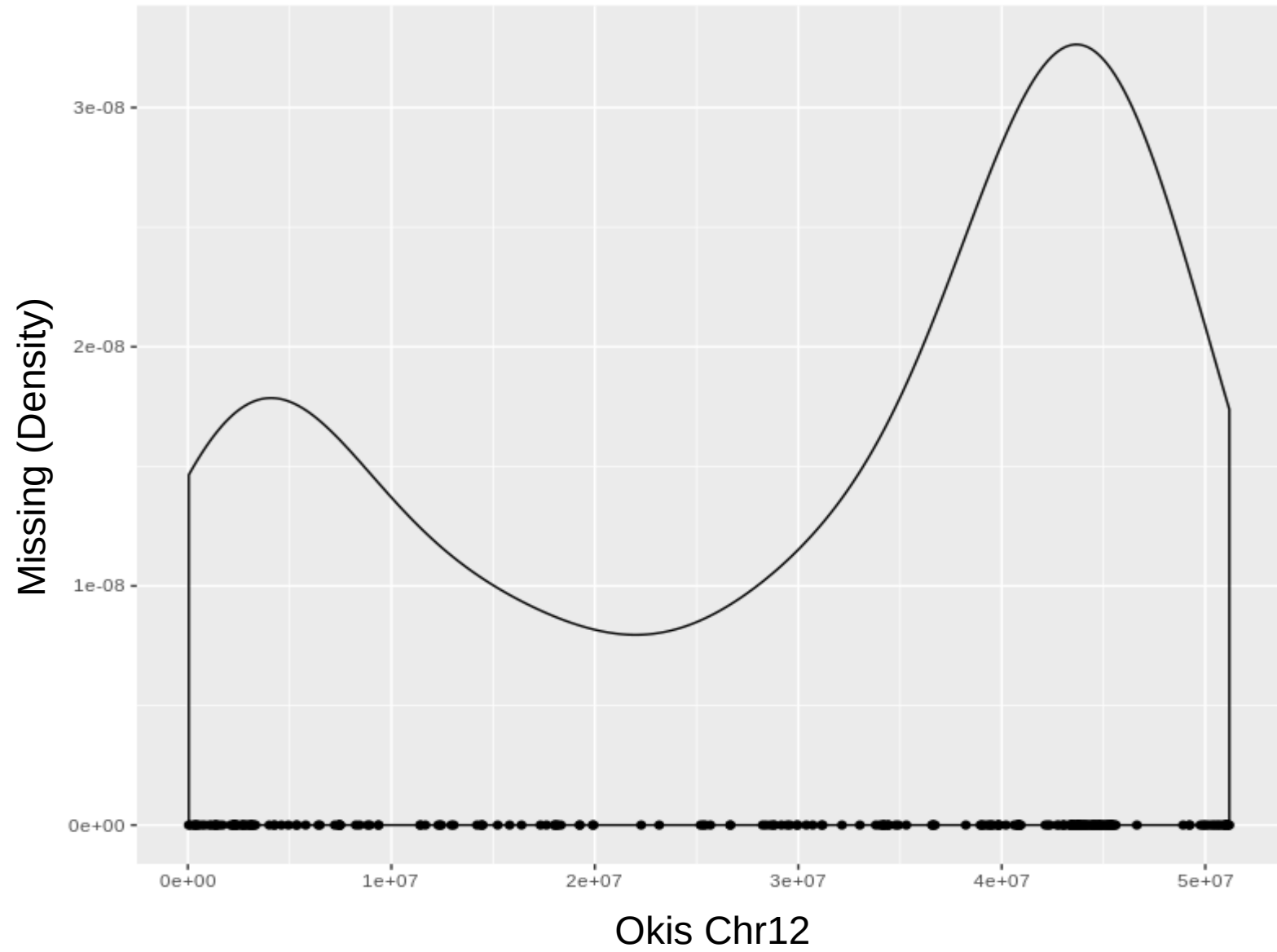

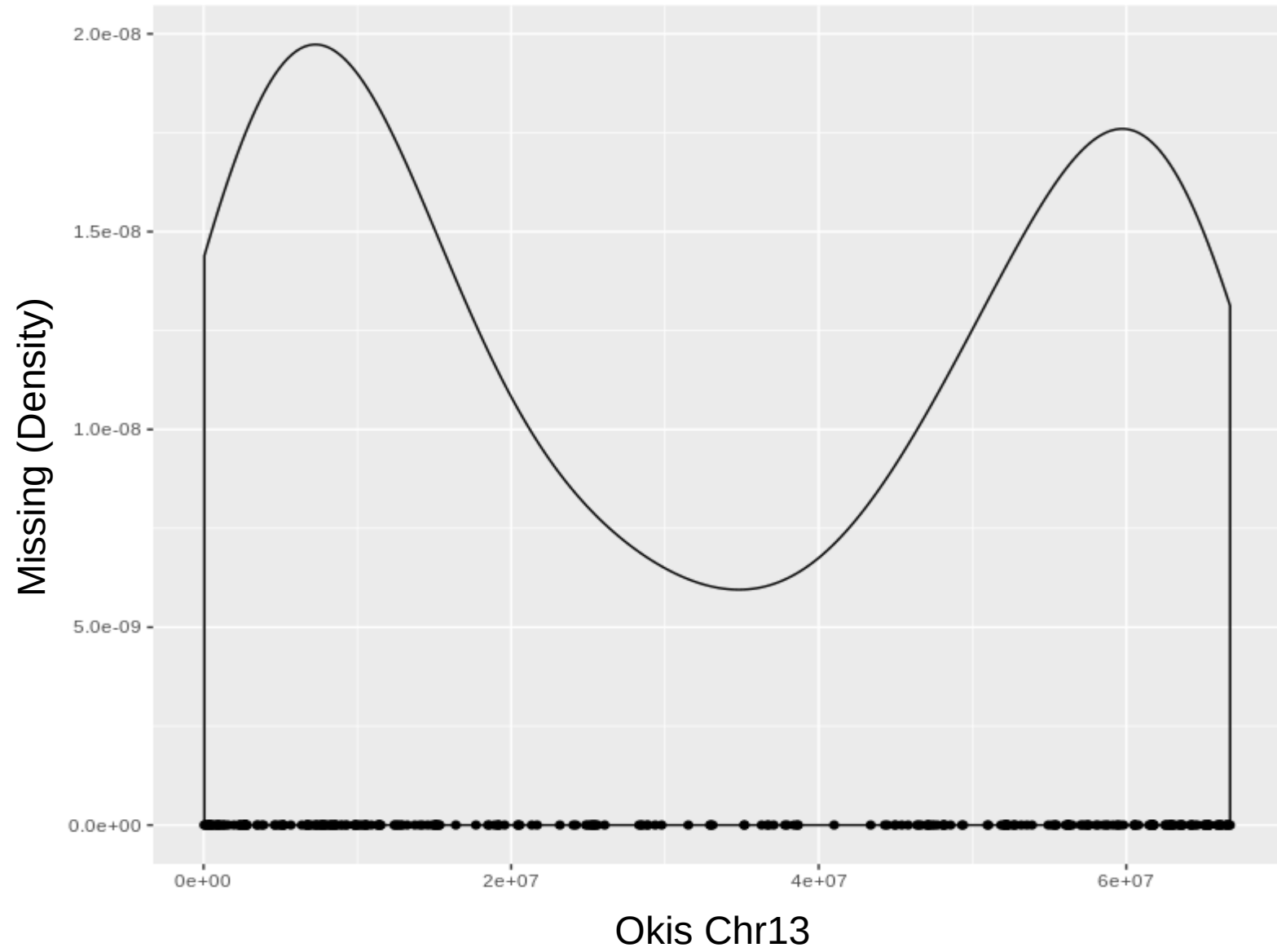

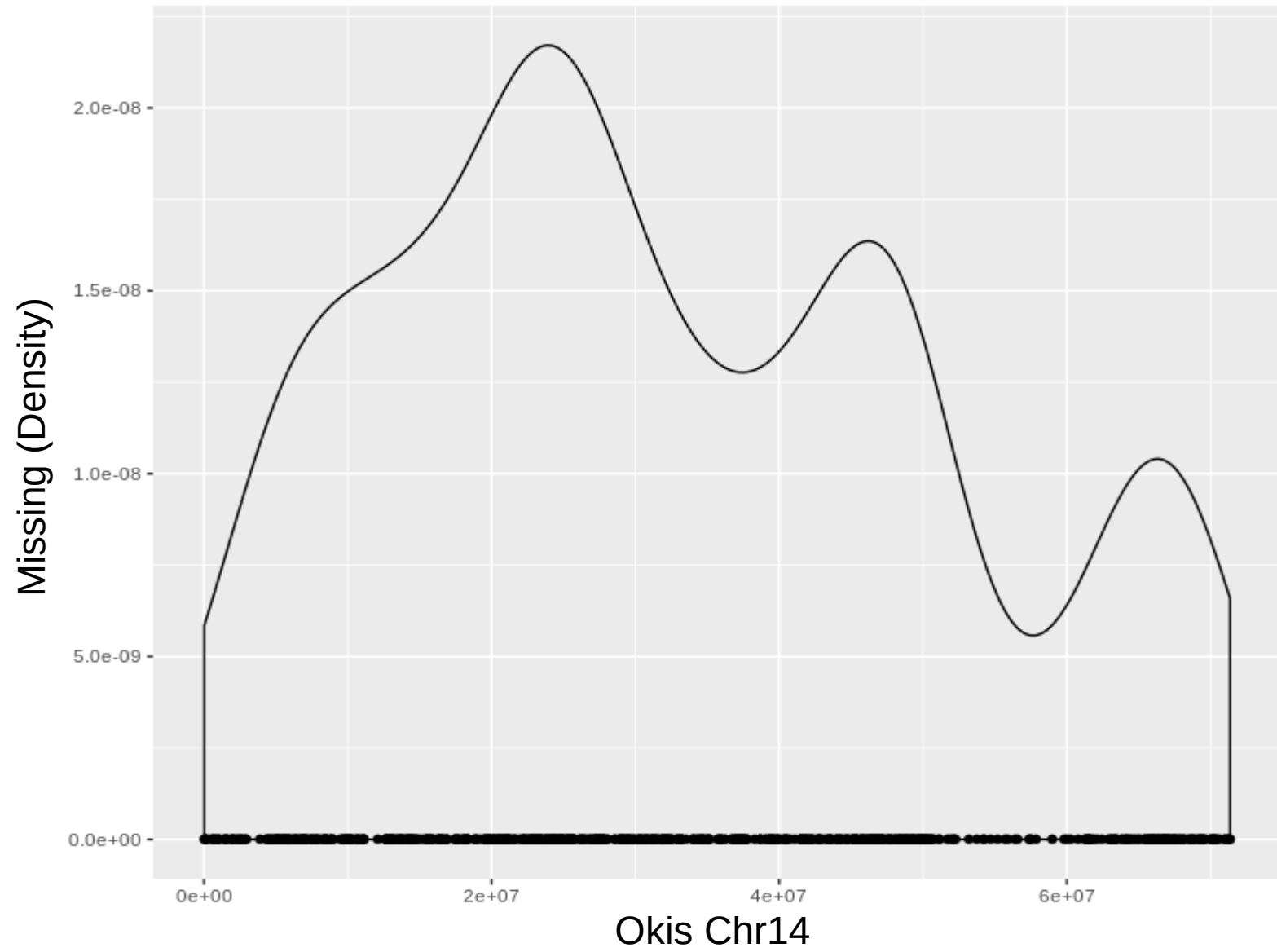

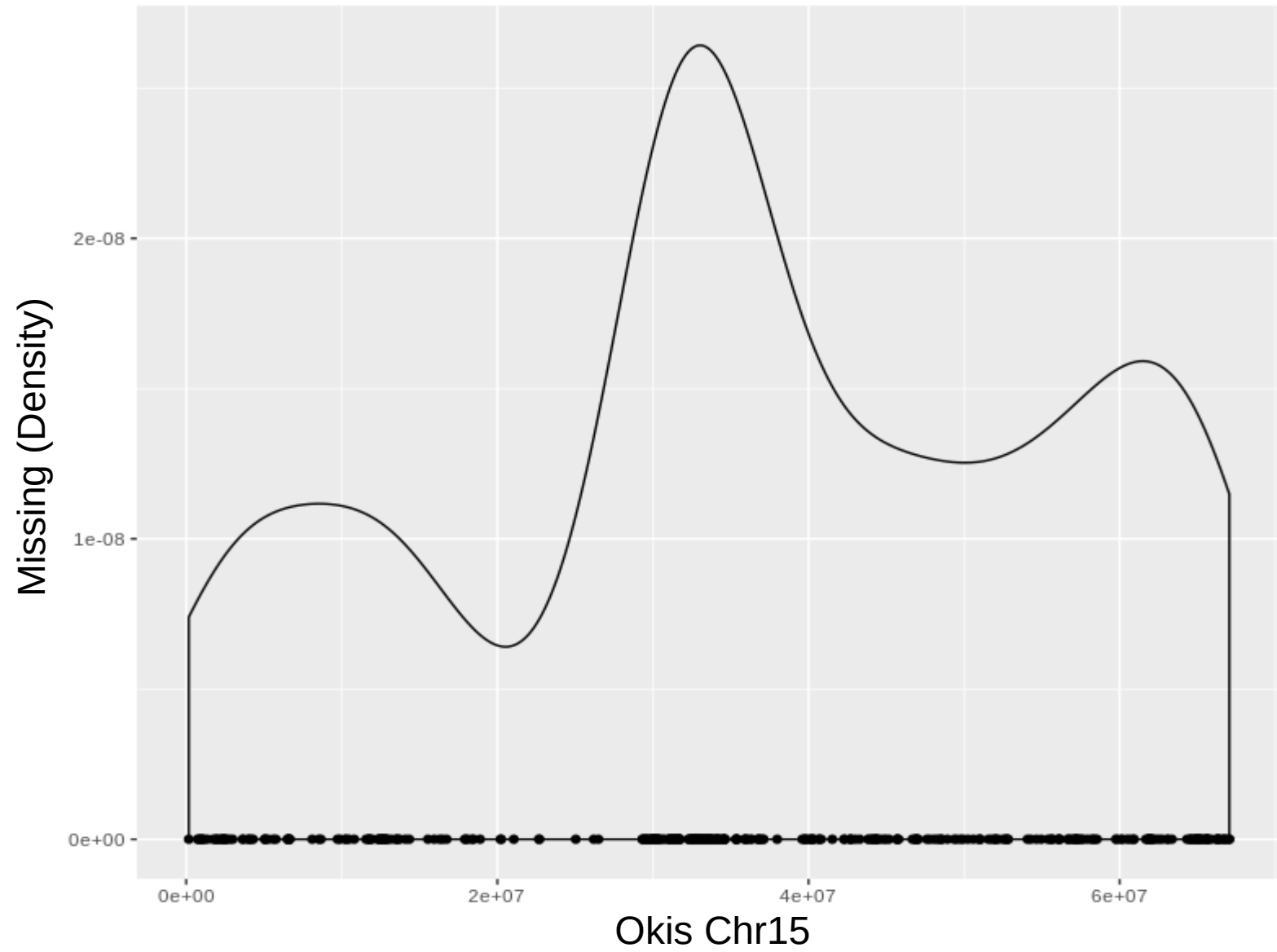

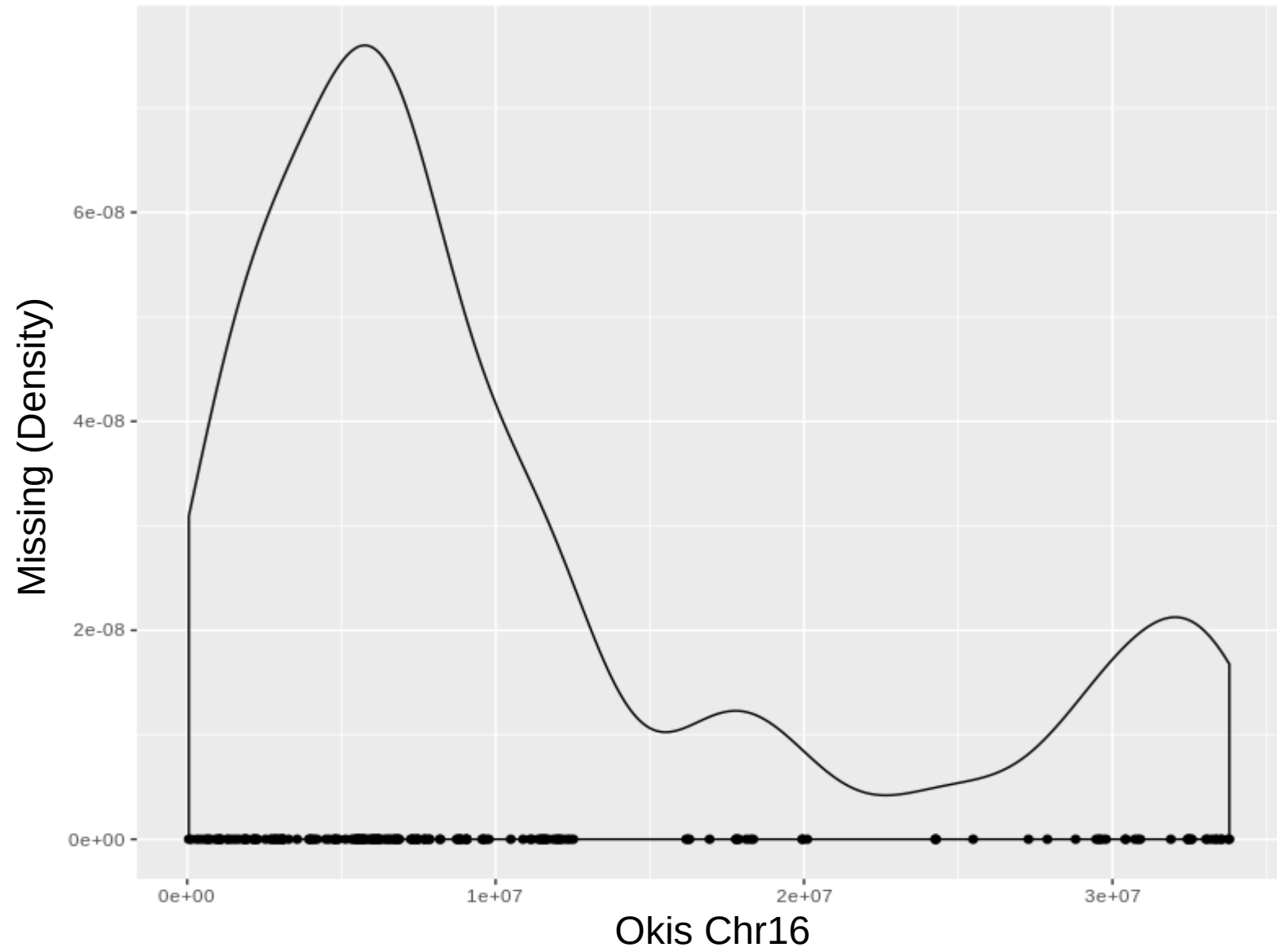

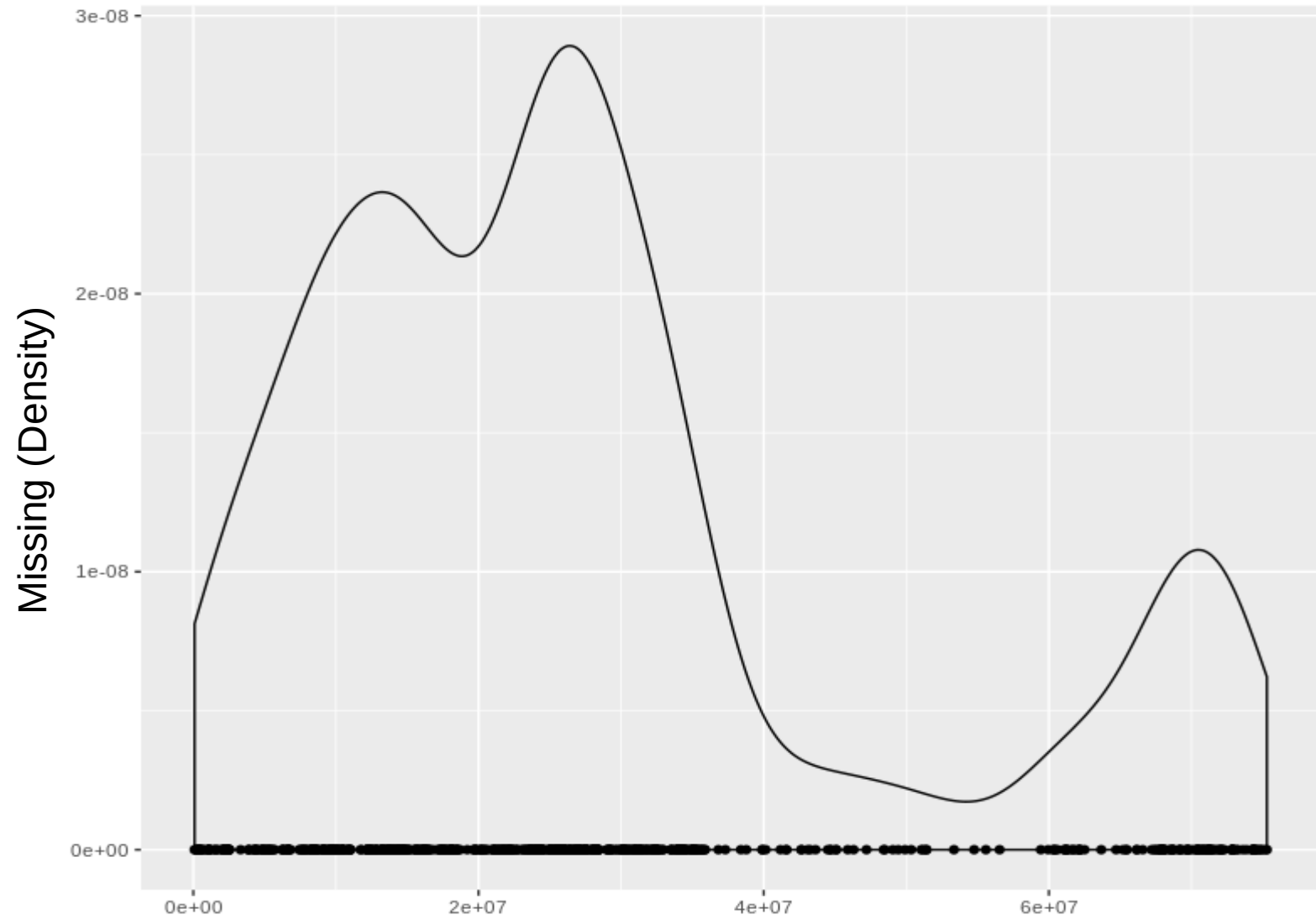

Okis Chr17

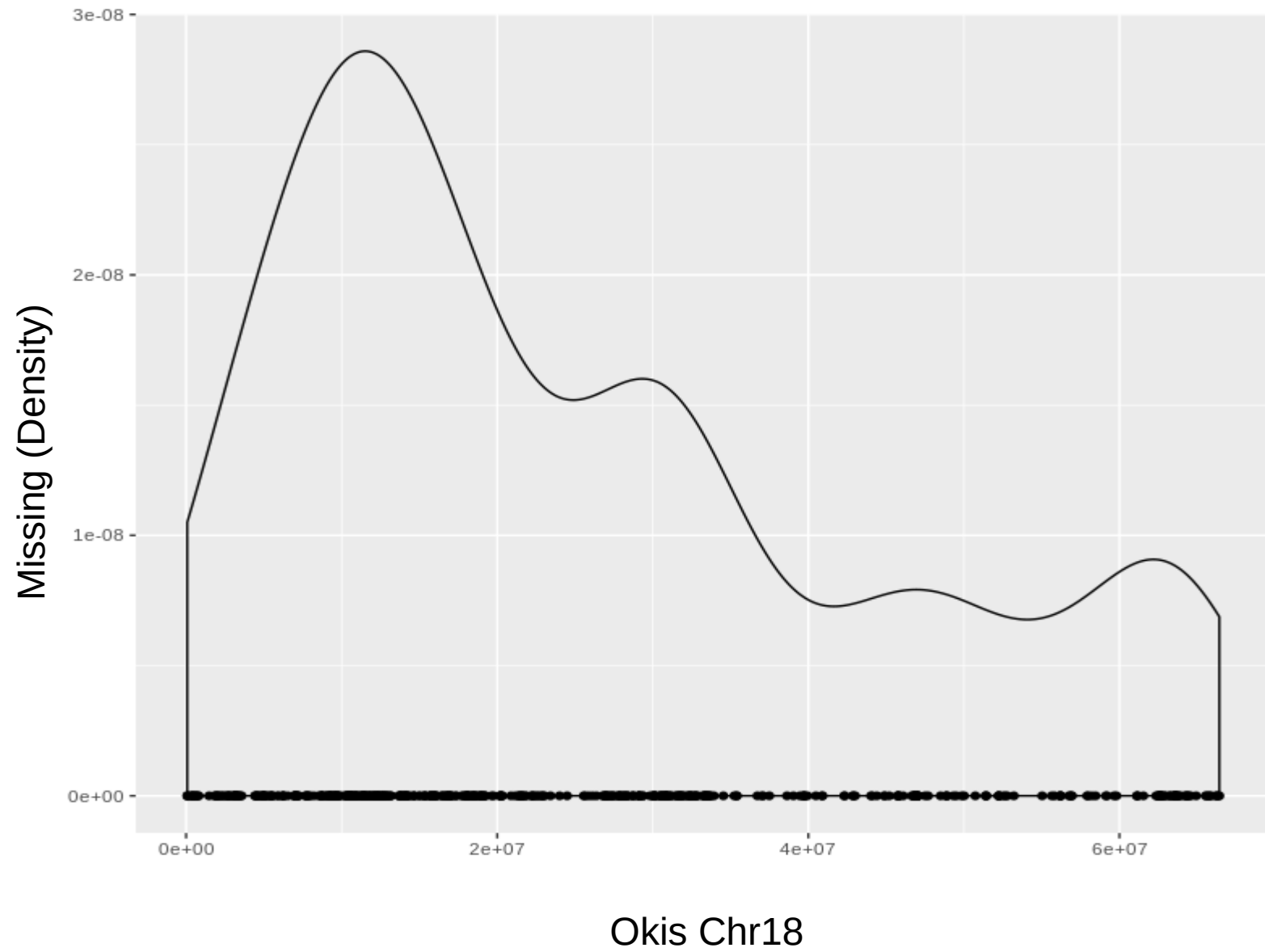

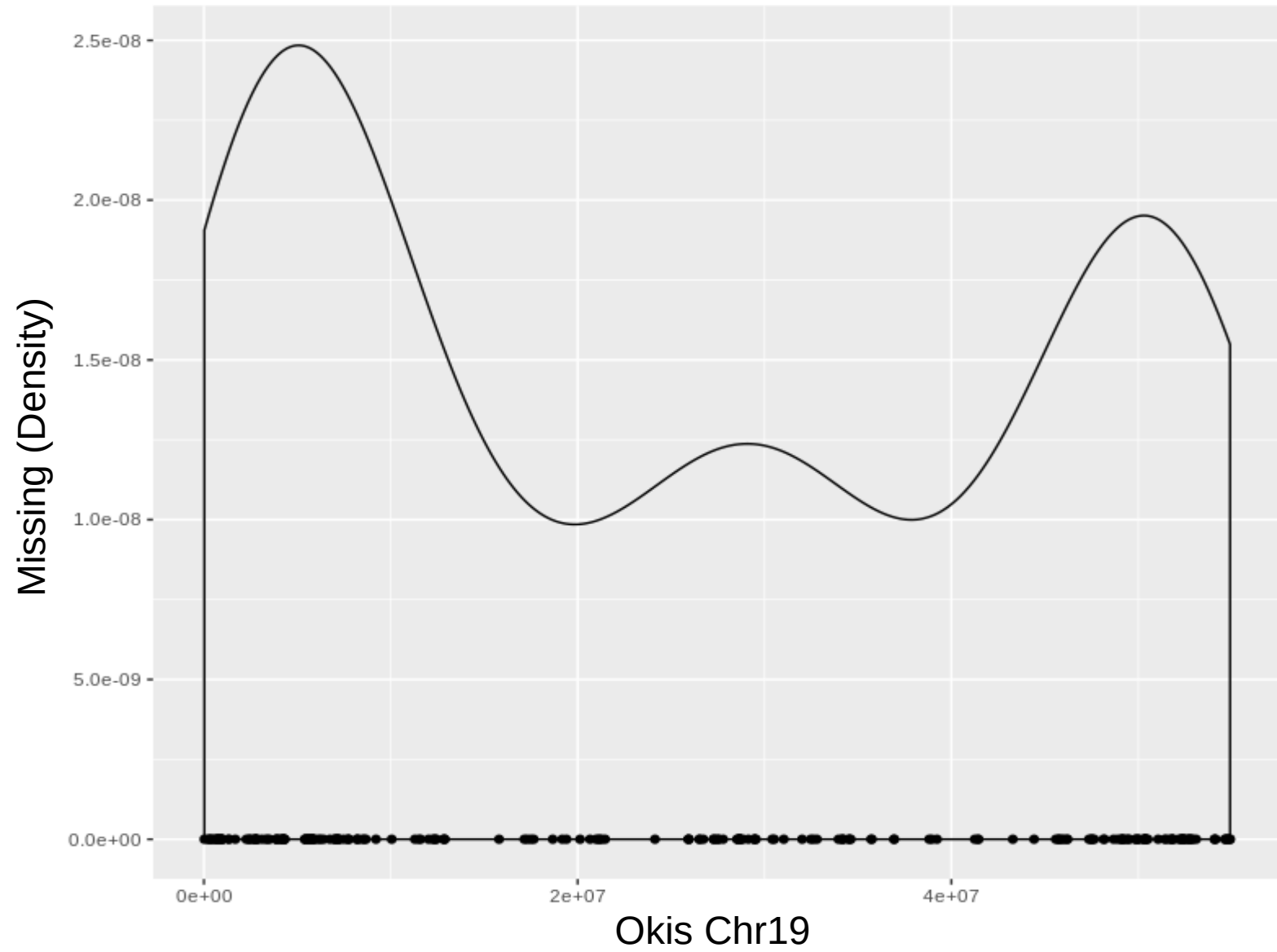

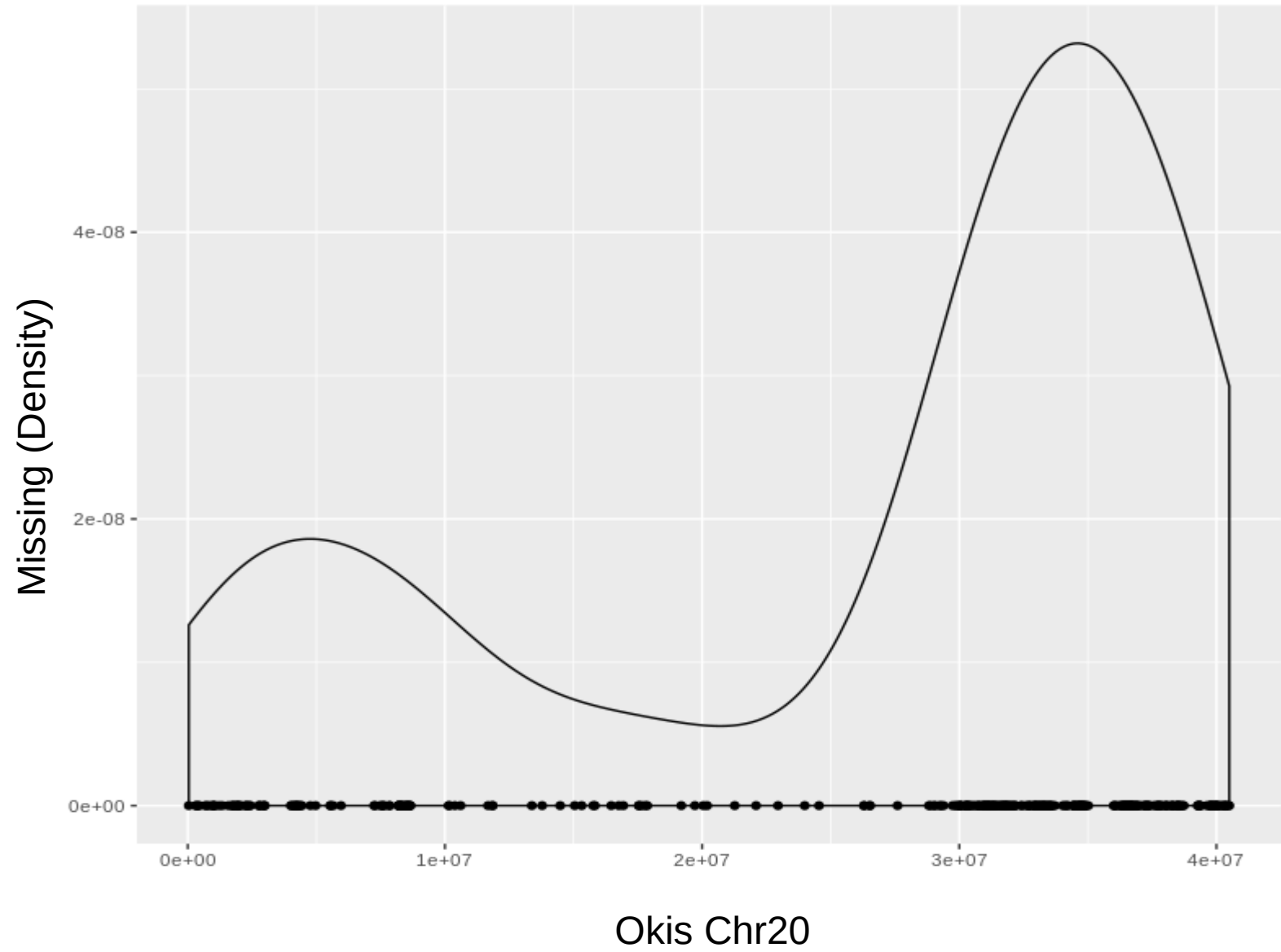

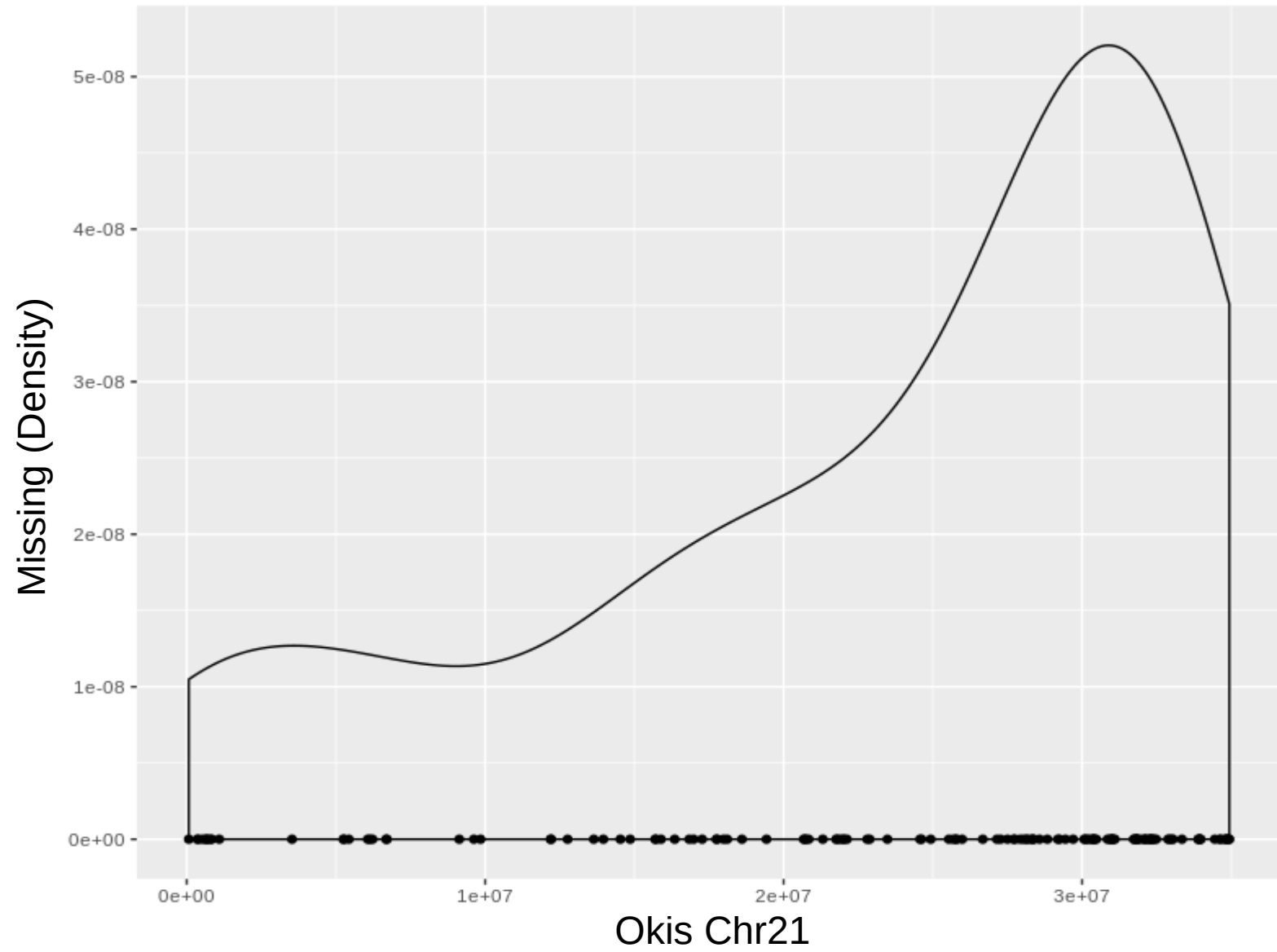

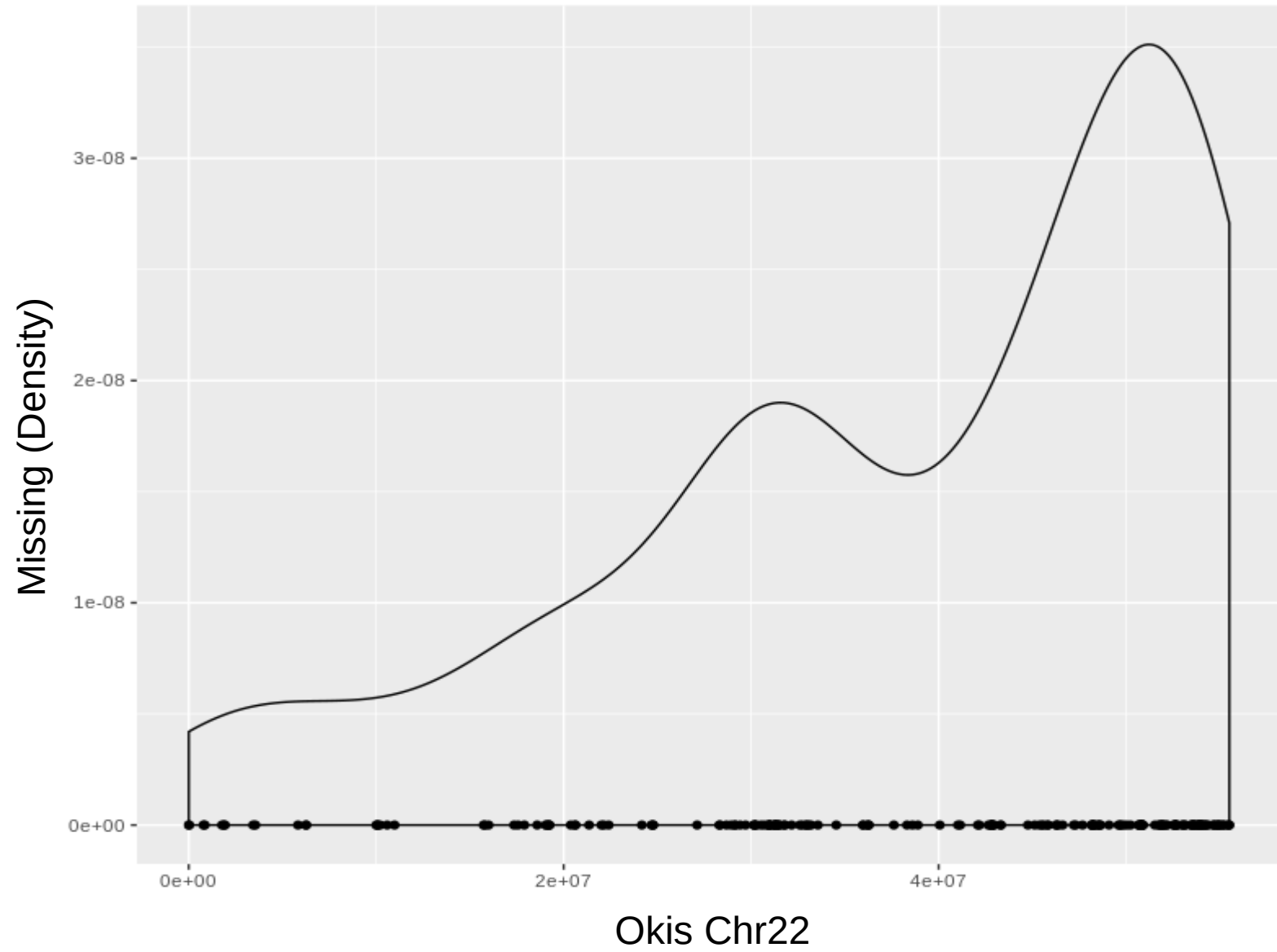

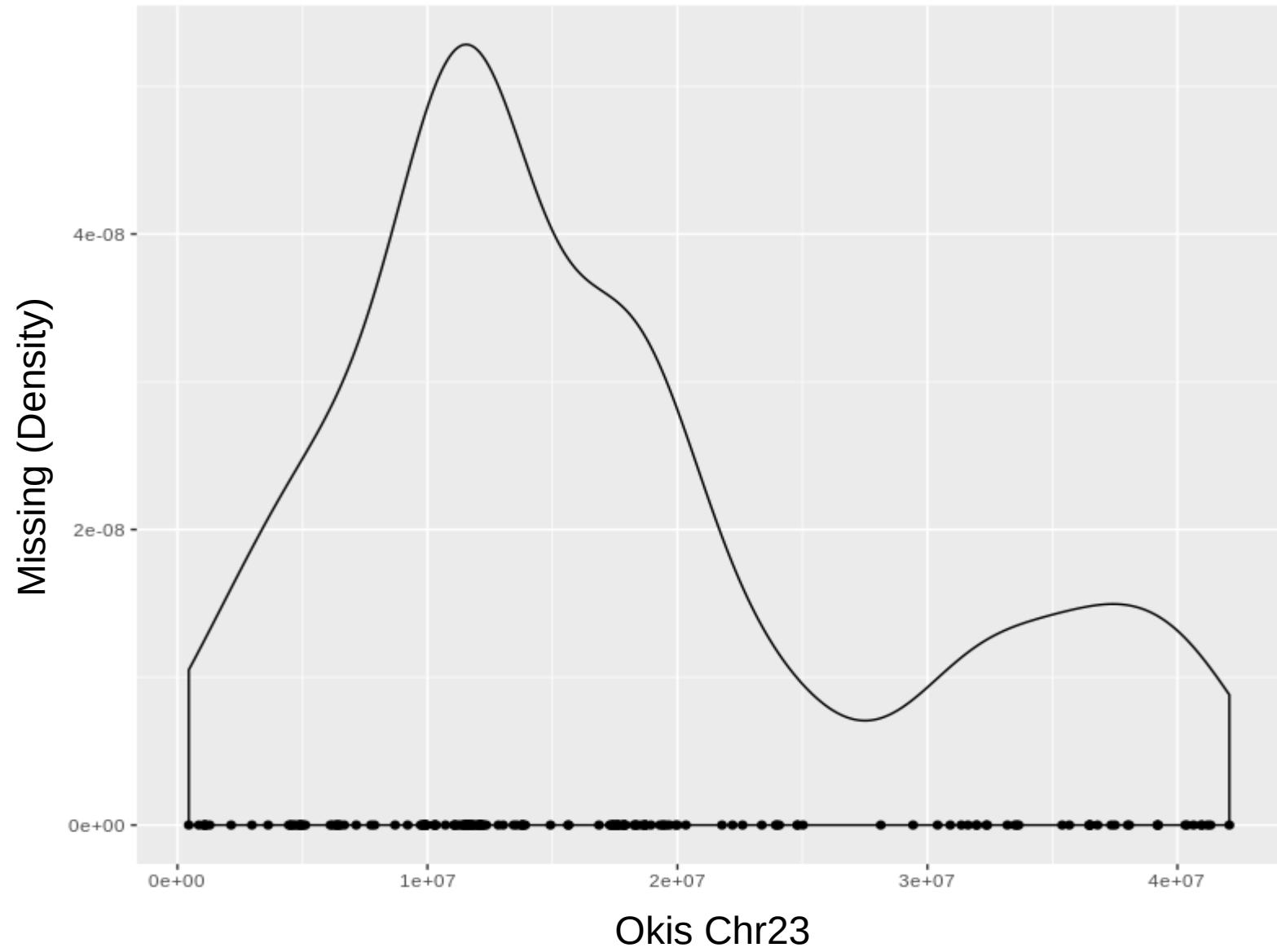

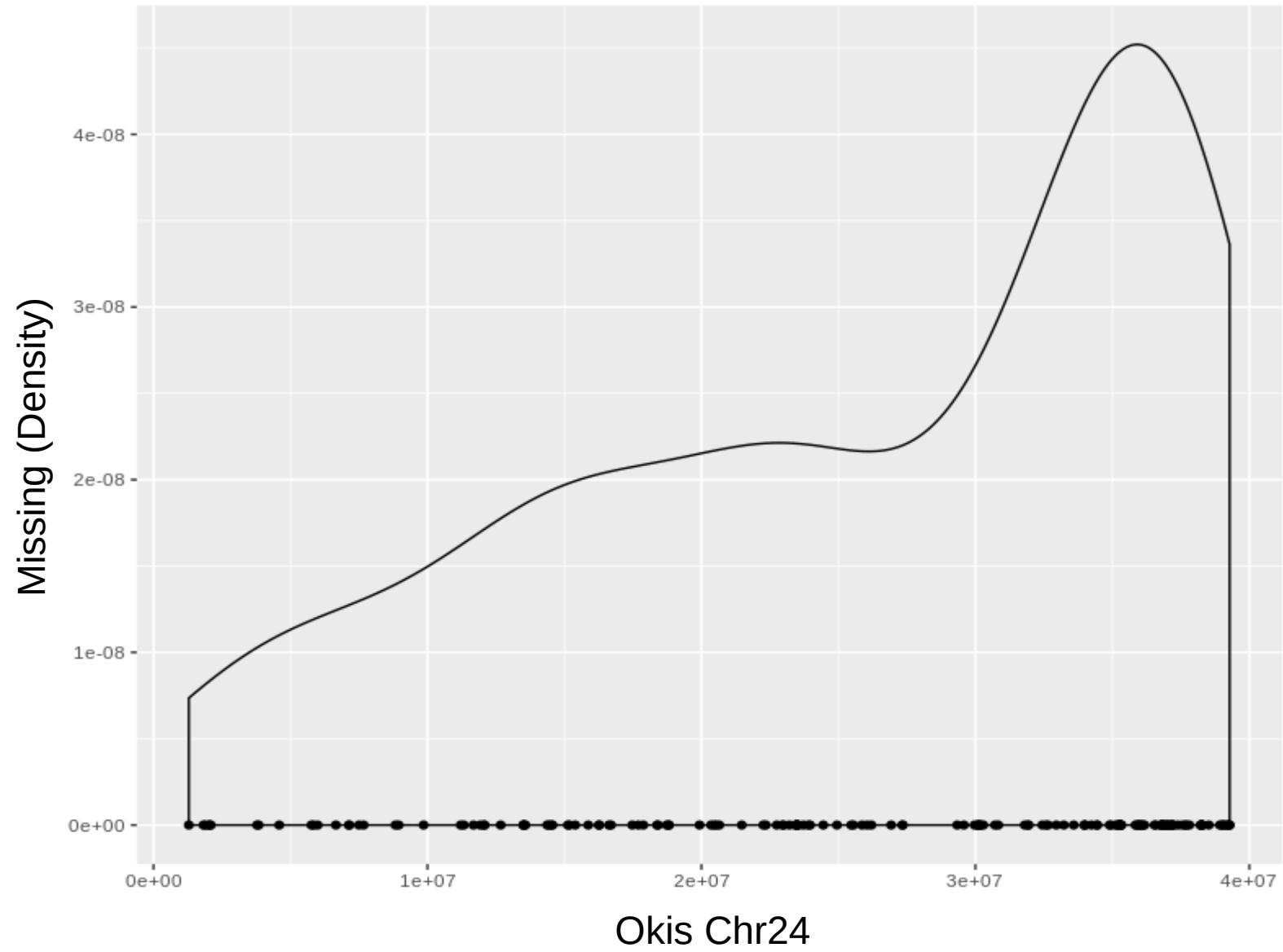

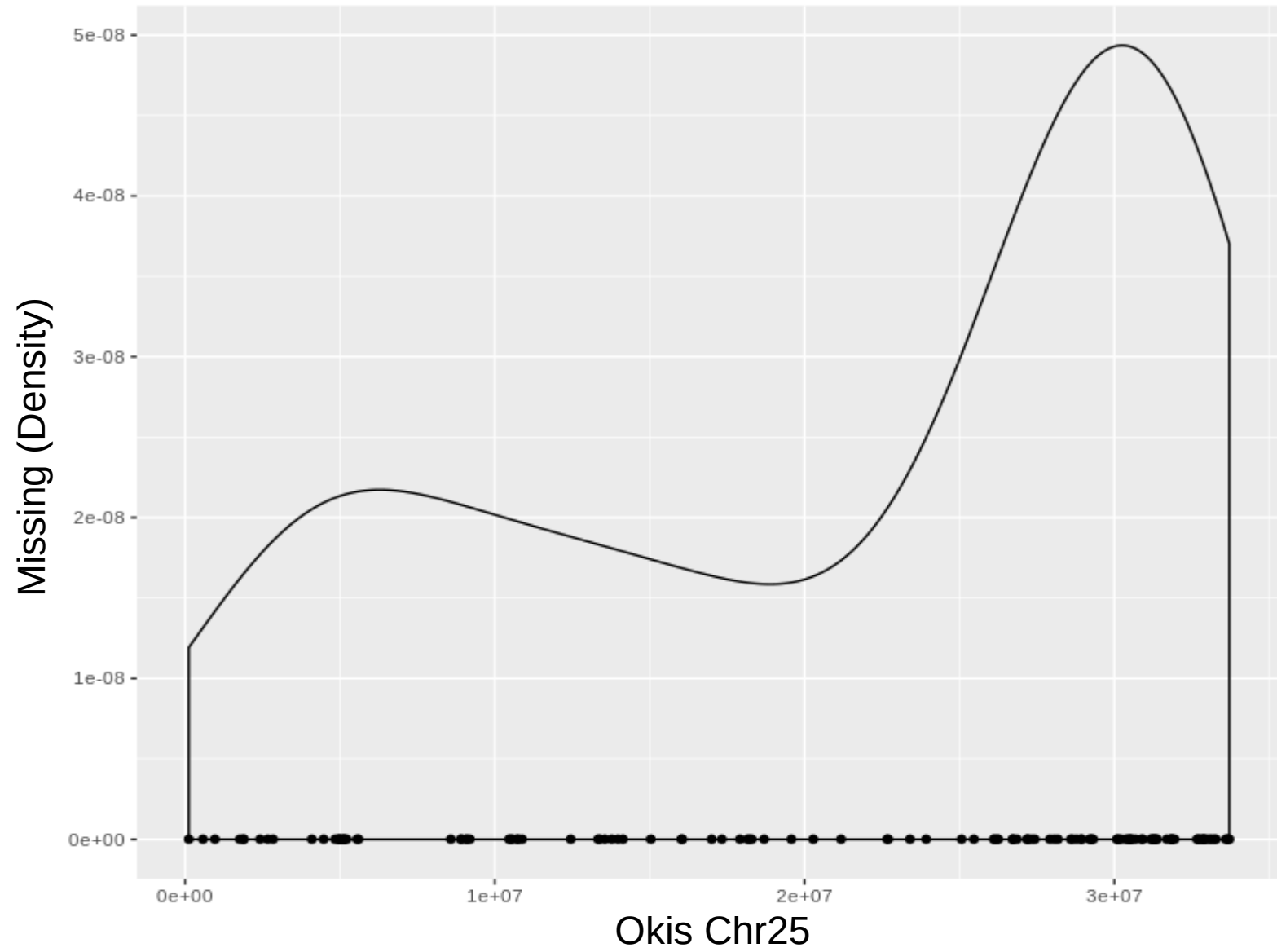

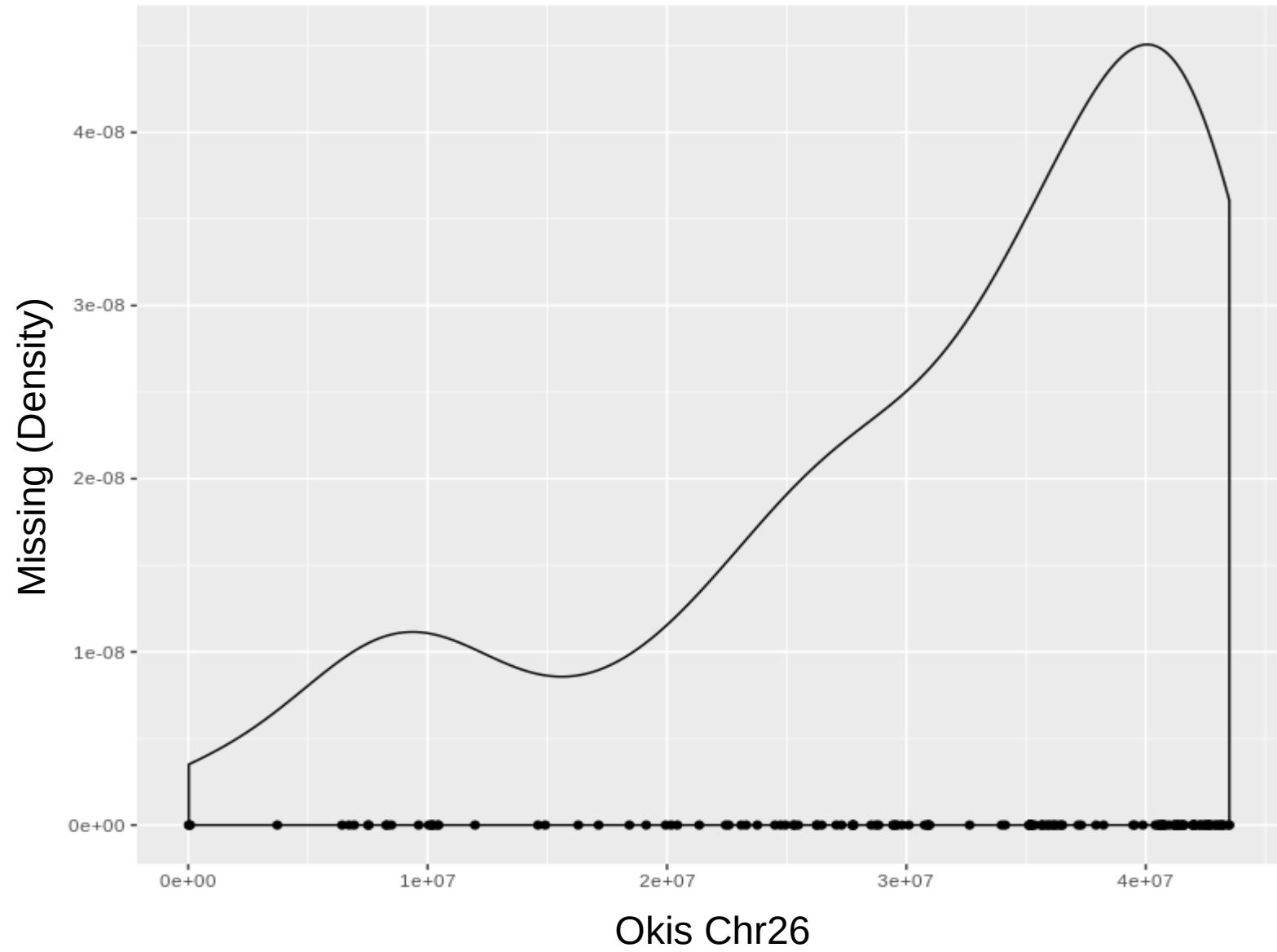

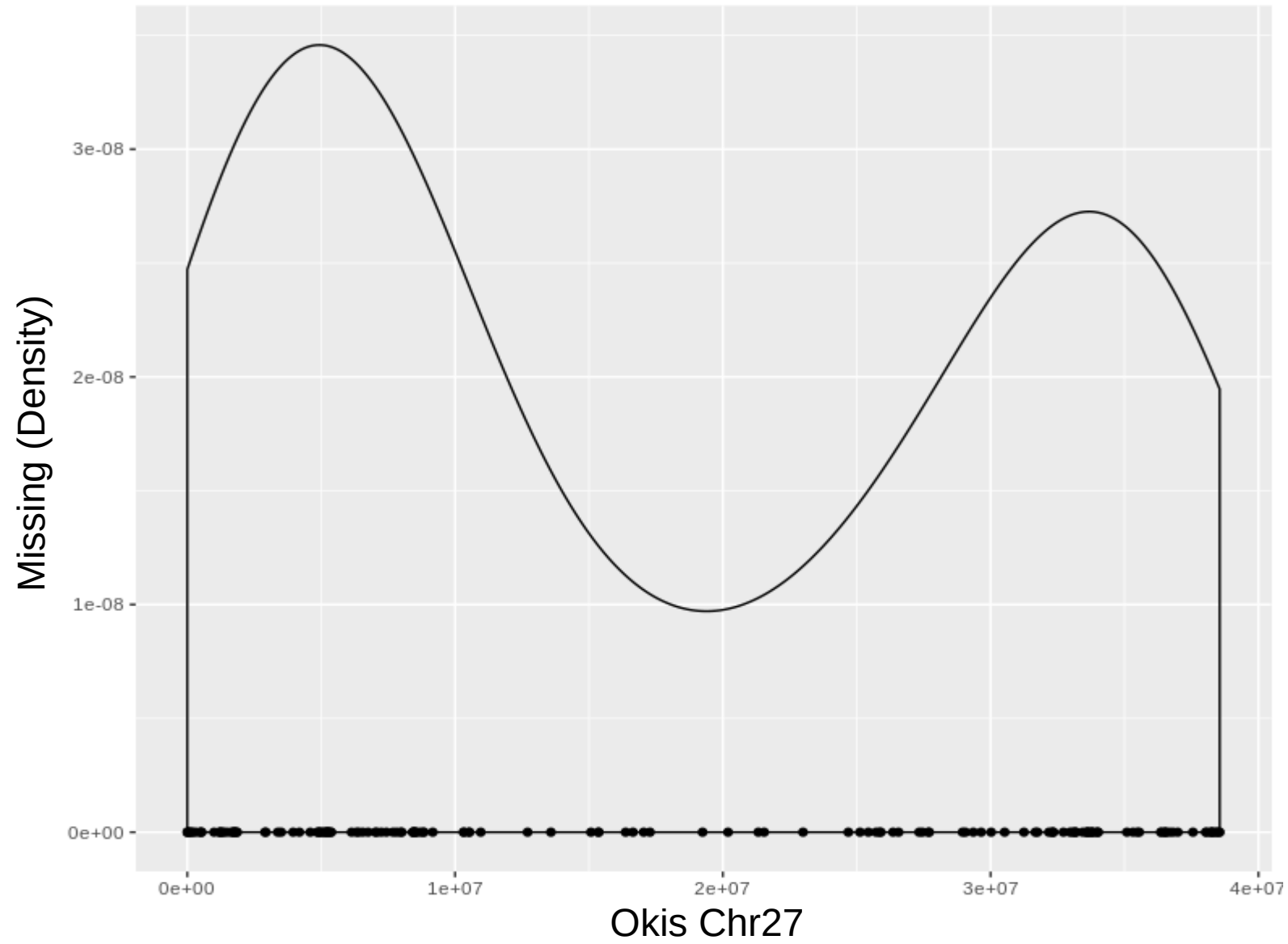

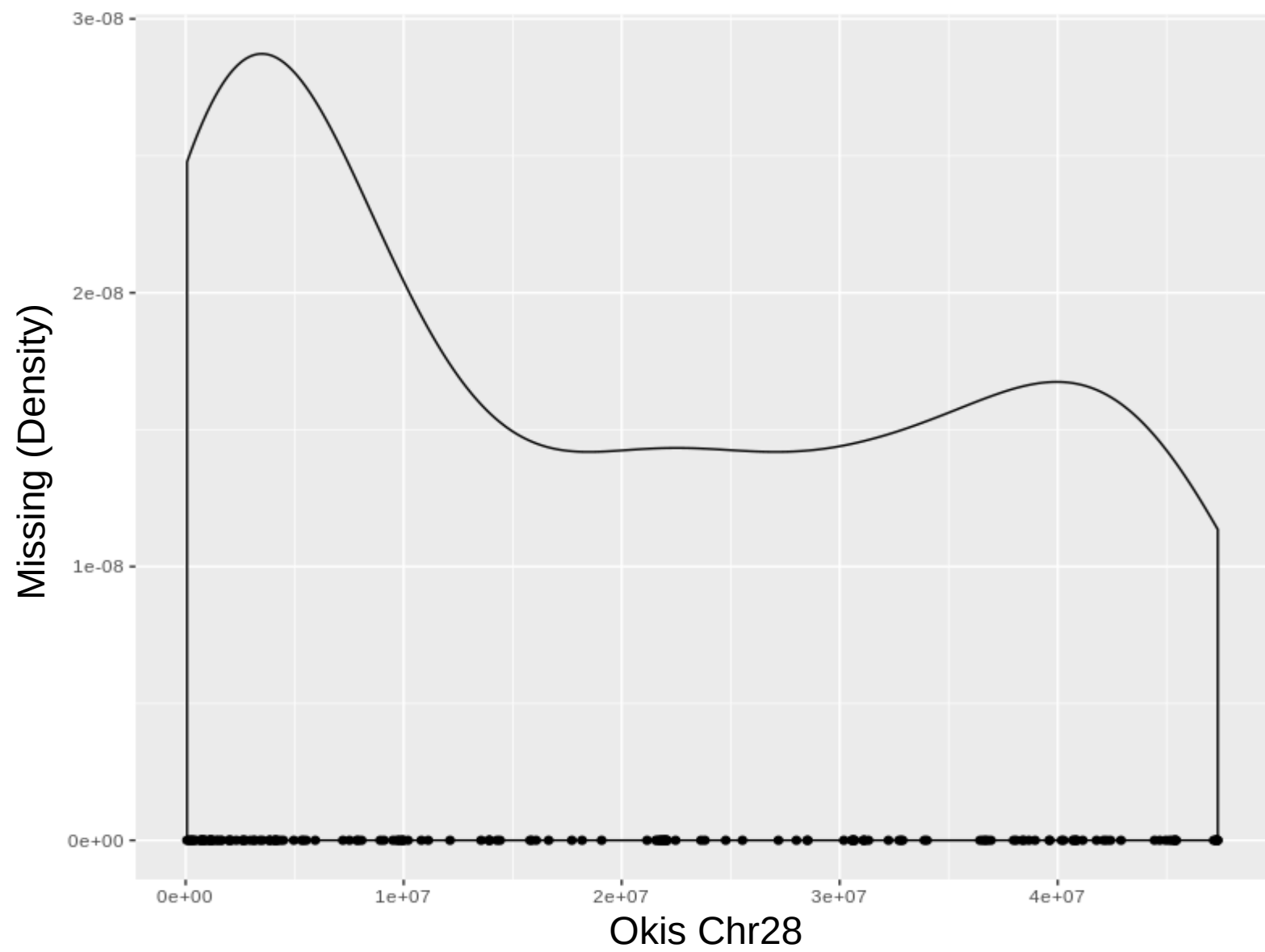

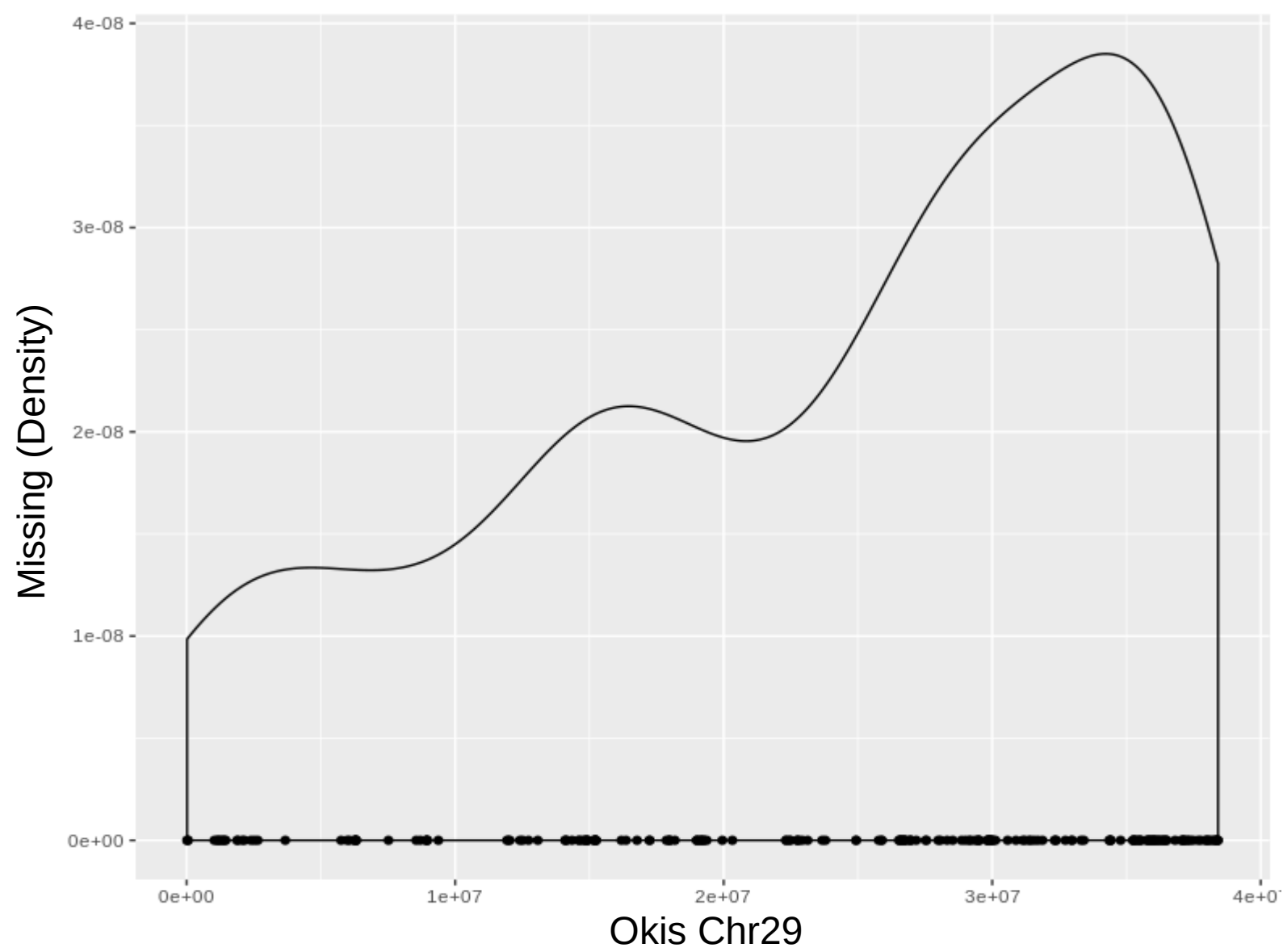

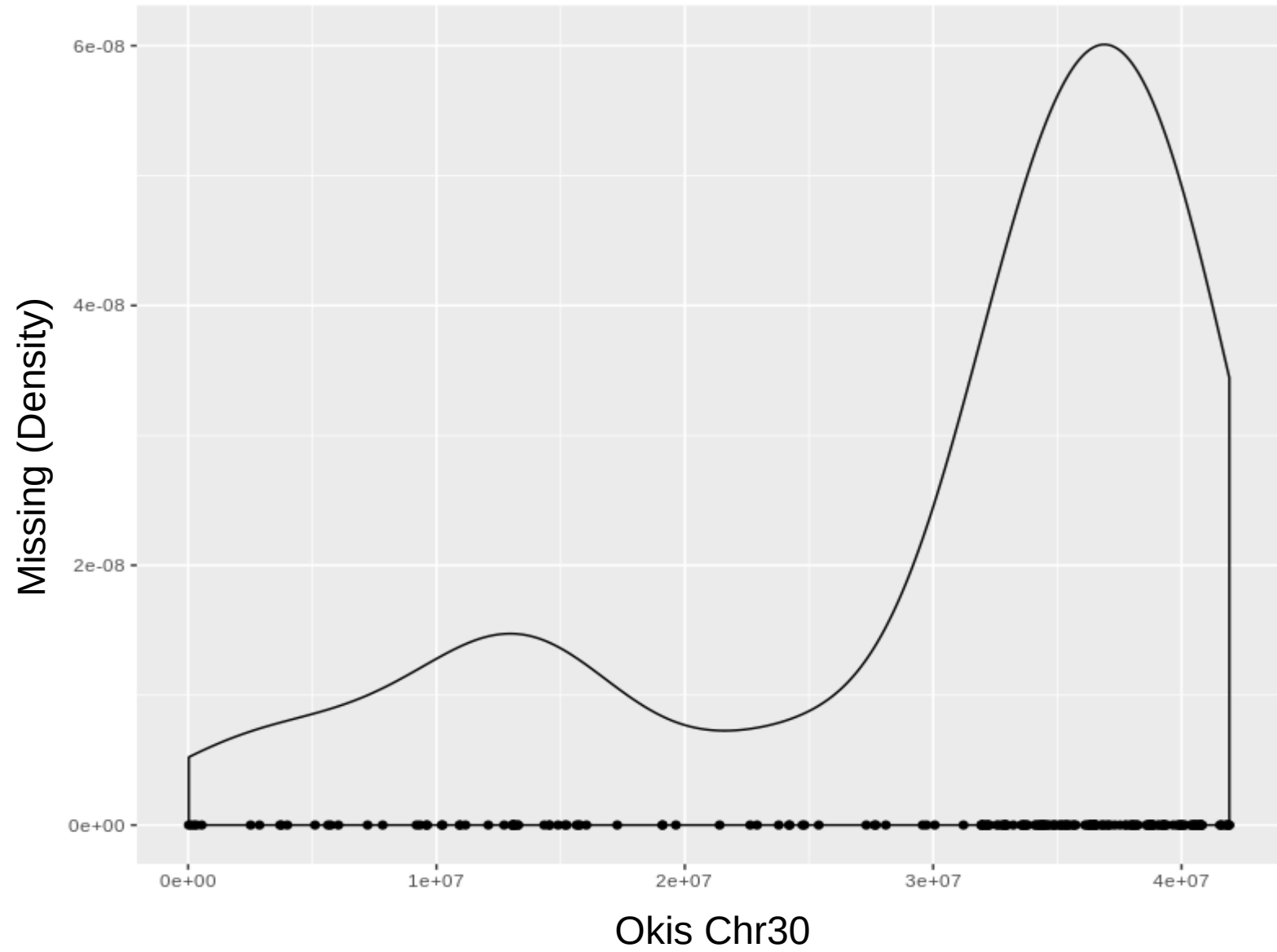

Supplement: S8 Fig — The x-axis is positions along the chromosome and the points represent the start position of a “missing” gene. The y-axis is the density of missing genes along the chromosome. (PDF) [file pone.0240935.s008.pdf]
