# Supplementary material for: The sockeye salmon genome, transcriptome, and analyses identifying population defining regions of the genome
Source: PLoS One. 2020 Oct 29;15(10):e0240935. doi: 10.1371/journal.pone.0240935 (PMC7595290; doi:10.1371/journal.pone.0240935)
Supplement: S1 Methods — (DOCX) [file pone.0240935.s012.docx]

## Samples

Pitt Lake sockeye salmon siblings (internally named: On170113-E2 for the individual used to generate the genome assembly (age: ~6 months), and On170719-1 used to generate transcriptome data (age: ~11 months)) were reared at the Fisheries and Oceans Canada facilities at Inch Creek, BC, Canada. The Canadian Council on Animal Care guidelines (CCAC, Animal Use Protocol Ex.7.1, Pacific Region Animal Care Committee management procedure 3.7) were followed as required by the Canadian government. These salmon were reared in 170 L tanks that were supplied with aerated well-water (10 ± 0.5°C) at a density of less than 5 kg/m^3^, with a natural photoperiod. The salmon were euthanized in a bath of 100 mg/L tricaine methanesulfonate buffered with 200 mg/L sodium bicarbonate. Multiple tissue samples (red muscle, hind gut, stomach, ovaries, gill, spleen, pituitary, white muscle, pyloric caeca, adipose, heart, liver, brain, mid gut, left eye, upper jaw, head kidney, and lower jaw) for RNA-seq/transcriptome generation were taken from On170719-1 (female, 14.0 g, 10.8 cm) by team dissection and stored in RNAlater Stabilization Solution as recommended by the manufacturer (ThermoFisher) until libraries could be prepared.

To generate genome resequencing data, samples (Table 1, S1 Table) were obtained from five sources: 1) Fisheries and Oceans Canada (various researchers), 2) Eric Taylor at the University of British Columbia, 3) Michael Russello at the University of British Columbia, 4) Scott Pavey at the University of New Brunswick, and 5) Theresa Godin at the Freshwater Fisheries Society of British Columbia. The locations of all the bodies of water that the samples were taken from can be found in Table1, S1 Fig., and S1 Table. Samples from Fisheries and Oceans Canada and Freshwater Fisheries Society of British Columbia were collected under the same procedure/permit as that above for the fish used for the genome assembly and transcriptome generation (see above for euthanasia protocol) except those taken from the Clearwater Trout Hatchery, which were non-lethally sampled following anesthesia in a tricaine methanesulfonate bath. Kokanee samples provided by Michael Russello were originally collected in partnership with the British Columbia Ministry of Forests, Lands and Natural Resource Operations, and in accordance with the University of British Columbia animal care protocol #A11-0127, and were sampled from operculum punches after fish had died naturally during the spawning season. Samples collected by Eric Taylor were collected under University of BC Animal Care and Use permits issued following CCAC protocols. Fin clips were non-lethally sampled after anesthesia in a tricaine methanesulfonate bath. Samples from Scott Pavey were sampled for a previous study and followed the same euthanasia protocol as Fisheries and Oceans Canada (1). All fish collected were either from wild or feral populations except for nine Columbia River drainage kokanee from Freshwater Fisheries Society of British Columbia (noted in S1 Table).

**Table 1. Sampling locations and information.**

| **Body of Water** | **Drainage** | **Ecotype** | **Latitude*** | **Longitude*** | **Samples** |
| --- | --- | --- | --- | --- | --- |
| Klukshu | Alsek | Sockeye | 60.29 | 223.00 | 4 |
| Albert Johnson | Aniakchak | Sockeye | 56.79 | 202.24 | 4 |
| Snootli | Bella Coola | Sockeye | 52.38 | 233.45 | 5 |
| Clearwater Trout Hatchery | Columbia | Kokanee | 51.65 | 239.94 | 5 |
| Columbia | Columbia | Kokanee | 50.30 | 244.14 | 4 |
| Hill | Columbia | Kokanee | 50.68 | 242.19 | 4 |
| Kootenay | Columbia | Kokanee | 49.55 | 243.18 | 7 |
| Meadow | Columbia | Kokanee | 50.25 | 243.01 | 14 |
| Norbury | Columbia | Kokanee | 49.47 | 244.53 | 4 |
| Okanagan | Columbia | Sockeye | 49.51 | 240.40 | 4 |
| Whatshan | Columbia | Kokanee | 50.09 | 241.90 | 4 |
| Adams | Fraser | Sockeye | 50.89 | 240.45 | 6 |
| Chilko | Fraser | Sockeye | 51.62 | 235.86 | 10 |
| Cultus | Fraser | Sockeye | 49.08 | 238.02 | 6 |
| Little Horsefly | Fraser | Kokanee | 52.37 | 238.67 | 4 |
| Pitt | Fraser | Sockeye | 49.32 | 237.32 | 7 |
| Tachie | Fraser | Sockeye | 54.66 | 235.23 | 5 |
| Takla | Fraser | Sockeye/Kokanee | 55.70 | 233.75 | 4/4 |
| Upper Horsefly | Fraser | Sockeye | 52.40 | 239.32 | 5 |
| Elovka | Kamchatka | Sockeye | 56.40 | 160.64 | 2 |
| Kronotskoye | Kronotskaya* | Kokanee | 54.77 | 160.23 | 4 |
| Tazimina | Kvichak | Sockeye | 59.96 | 205.16 | 4 |
| Woody Island | Kvichak | Sockeye | 59.76 | 205.75 | 4 |
| Babine | Skeena | Sockeye/Kokanee | 55.70 | 232.30 | 4/4 |
| Shale | Skeena | Kokanee | 54.74 | 233.99 | 4 |
| Hansen | Wood | Sockeye | 59.32 | 201.30 | 4 |

*Locations are approximate to body of water

References

1. Pavey SA, Hamon TR, Nielsen JL. Revisiting evolutionary dead ends in sockeye salmon (Oncorhynchus nerka) life history. Can J Fish Aquat Sci. 2007 Sep 1;64(9):1199–208.
