# Supplementary material for: The sockeye salmon genome, transcriptome, and analyses identifying population defining regions of the genome
Source: PLoS One. 2020 Oct 29;15(10):e0240935. doi: 10.1371/journal.pone.0240935 (PMC7595290; doi:10.1371/journal.pone.0240935)
Supplement: Attachment — Submitted filename: Response to Reviewers.docx [file pone.0240935.s014.docx]

**Comments from reviewer 3**

Major comments
The only major comment I have concerns the length of the new version of the manuscript. I tried to find a section or two that could be moved to supplemental, but the only good candidate was the sampling details on lines 93-129 (as well as table one).

**The sample section was moved to S1 Methods.**

Minor comments
Lines 53-54: start the sentence with “This split between…” and then add “suggests” between “salmon species and” and “two common North American…”

**This was changed.**

Line 57: add “the phenotype is” to the text within brackets, “(i.e., the phenotypes are polyphyletic)”
I think adding some text to line 58 might help the reader understand why the Fraser and Colombia Rivers are different to the rest of the sockeye range. Perhaps something like “where multiple populations of Kokanee are more closely related to each other than sympatric sockeye salmon”.

**This was changed.**

Line 140: add how large the size selected fragment of DNA was.

**Added that the peak was around 488 bp.**

Line 174: add the parameters used for filtering with FastQC.

**Added that default settings were used.**

Paragraph titled “clustering and chromosomal variation underlying population structure
This paragraph is very interesting, but the important points get a bit lost as the reader tries to keep which population is which straight. I’d suggest re-writing this section perhaps to emphasize that what’s interesting here is the discrepancy in the normal dogma that sympatric kokanee and sockeye are more closely related to each other than either is to allopatric populations of the same ecotype. Perhaps, the authors should start by saying “There are three clusters, one of which was composed of samples from the upper Columbia” and then go on to talk about the discrepancy between studies with regard to some kokanee forming a monophyletic group.

**Changed to make this result more clear.**

Lines 750-753: the information in brackets could be deleted as the methods used are well explained in the methods section.

**Removed.**
